# Blended therapy for adolescents with chronic health conditions to increase fatigue-related self-efficacy (Booster): protocol for a single-case multiple baseline study

**DOI:** 10.1186/s13063-025-08960-1

**Published:** 2025-07-24

**Authors:** Maartje D. Stutvoet, Anouk Vroegindeweij, Jan Houtveen, Raphaële R. L. van Litsenburg, Elise van de Putte, Remco C. Veltkamp, Sanne L. Nijhof

**Affiliations:** 1 Department of Paediatrics, Wilhelmina Children’s Hospital, University Medical Centre Utrecht, Utrecht University, Utrecht, The Netherlands; 2 Princess Máxima Centre for Paediatric Oncology, Utrecht, The Netherlands; 3 Department of Information and Computing Sciences, Utrecht University, Utrecht, The Netherlands

**Keywords:** Fatigue, Self efficacy, Early medical intervention, Experience sampling, Single-case experimental design, Mobile health, Lifestyle, Chronic disease, Adolescent

## Abstract

**Background:**

Fatigue is a common symptom in adolescents with a chronic health condition. Persistent fatigue and its impairments may be prevented by early intervention with Booster. Booster is a transdiagnostic blended care intervention that aims to increase fatigue-related self-efficacy (FSE). Through experience sampling methodology (ESM) via the Booster smartphone app, Booster helps users gain personalised insight into the relationship between their thoughts, feelings, activities, and fatigue. Based on this insight and shared-decision making, the participant and executive investigator set personal lifestyle goals, such as more exercise and fewer daytime naps. The previous version of Booster, PROfeel, has already been shown effective in treating persistent fatigue in youth. Booster’s value as an early intervention has yet to be studied. To better suit this aim and align with user preferences, the new Booster app includes features like goal attainment assistance, daily outcome tracking, and motivating game mechanics (e.g., rewards and a minigame). This protocol describes a study to examine the effect of Booster on FSE and the other study outcomes fatigue, school participation, life satisfaction, and perceived health. The secondary aim is to explore individual differences regarding (moment of) changes in outcomes during the Booster intervention.

**Methods:**

It is a single-centre study with a multiple baseline single-case experimental design (SCED). We aim to include twenty adolescents ages 12 to 18 years with a chronic health condition and fatigue. Booster’s effect on outcomes will be measured with a daily survey during Phase A (baseline) and B (intervention). The start of phase B will be randomised across cases. The effect will be assessed with the multiple baseline single-case randomisation test (SCRT) at the group level. Additionally, at the single-case level, we will explore change using permutation distancing tests (PDTs), single-case interrupted time series analysis (ITSA), and change point analyses. Also, we will assess participant characteristics associated with (long-term) improvements.

**Discussion:**

Booster uses innovative methods by combining tailored ESM-insight, mHealth and healthcare professional support. Group-level analysis, strengthened by single-case observational analyses, will evaluate the effectiveness of ESM-supported blended care as an early fatigue intervention and identify its potential working mechanisms. It lays the groundwork for implementing ESM tools in clinical practice.

**Trial registration:**

ClinicalTrials, NCT06562335. Registered on 24 July 2024, https://clinicaltrials.gov/study/NCT06562335.

**Supplementary Information:**

The online version contains supplementary material available at 10.1186/s13063-025-08960-1.

## Introduction

### Background and rationale {6a}

Over recent decades, advancements in paediatric healthcare have increased survival rates, leading to more children living with chronic conditions like autoimmune diseases or post-cancer treatment [1–3]. Growing up with a chronic health condition is challenging, as is shown by decreased social functioning [4, 5], delayed developmental milestones [6], and more mental health problems [4, 5, 7, 8]. Severe fatigue is a common and potentially disabling symptom. Prevalence ranges from 5 to 72%, depending on the condition and measurement instrument or method [9]. Despite this broad range, children with a chronic health condition consistently show more clinically relevant fatigue than their healthy peers [9]. Severe fatigue restricts participation in regular activities, lowers quality of life (QoL), and impacts psychosocial health [10–13]. The impact of fatigue and its treatment scarcity urged patients with inflammatory bowel disease (IBD) and juvenile idiopathic arthritis (JIA) to prioritise understanding and treating fatigue in their research agenda [14, 15]. Also in the oncology research field, improving the clinical management of fatigue has high priority [16].

Research indicates that severe fatigue is largely independent of disease activity, with either no [17, 18] or minimal associations found [19]. However, fatigue strongly correlates with transdiagnostic (i.e. generic) factors like support at home, physical activity, and depressive symptoms [20, 21]. The biopsychosocial model supports this, viewing fatigue as a result of the interplay of biological, psychological, and social factors [22, 23]. This complex interplay varies individually, suggesting no one-size-fits-all solution [24, 25]. Thus, a transdiagnostic approach is promising, [26, 27] in which universal mechanisms may be considered and targeted through personalised care [24, 25].

The significant burden of fatigue in children with a chronic health condition underscores the urgency for treating persistent severe fatigue. Even more, it emphasises the need for early interventions, specifically at a stage when fatigue-related impairments are still minimal or nonexistent. Early interventions could avert fatigue progression and prevent the development of these impairments. General self-efficacy is defined as the belief in one’s capabilities to influence events affecting their life [28]. It influences behaviour, resilience, and affect [28, 29]. Research shows self-efficacy predicts fatigue in children with JIA [18]. In youth with cancer [30] and survivors of childhood cancer [31], it was shown that self-efficacy influences QoL via fatigue. Additionally, a post-cancer physical activity intervention in children initially boosted self-efficacy, then reduced fatigue and improved QoL [32]. Therefore, we consider fatigue-related self-efficacy (FSE) a key target for early intervention.

A transdiagnostic tailored intervention that was successful in reducing fatigue severity in youth with severe persistent fatigue with a chronic health condition is PROfeel [33, 34]. PROfeel also boosted FSE in this group [33] and appeared most effective in youth with relatively less severe symptoms [34]. PROfeel combines an Experience Sampling Methodology (ESM)-based smartphone app with face-to-face feedback conversations. This blended care combination profits from the advantages of both on- and offline care. Patients completed a brief ESM survey multiple times a day to gain insight into the fluctuations of their fatigue and fatigue-related factors, as well as the underlying associations. Ultimately, via shared-decision making, patients formulated personal lifestyle goals to reduce fatigue severity and improve FSE and QoL. A similar approach may be useful as an early intervention strategy. To adapt PROfeel for early intervention, improvement areas were distilled from evaluations with patients, their parents, and healthcare professionals. In a participatory design process, the intervention was changed accordingly and renamed “Booster”. Booster is a transdiagnostic and tailored intervention for fatigue in children with a chronic health condition. Booster intends to increase FSE through personalised fatigue insight and subsequent lifestyle changes, supported by the Booster smartphone app and face-to-face care in consecutive intervention stages. By increasing FSE, the Booster intervention aims to prevent the progression of fatigue and its impairments.

This protocol outlines the overall research objectives of the Booster study, as well as the planned methods, analyses, and anticipated strengths and limitations. The focus of this protocol paper will be on describing the intervention design and the planned analyses at the group and single-case level. The Booster study combines phase III and phase II elements of the Medical Research Council framework for complex interventions [35]. It aims to test the efficacy of the Booster intervention in a new population (phase III) while also exploring potential mechanisms of action and user engagement (phase II). As such, this study represents an important step toward the future implementation of ESM-based tools in paediatric clinical practice.

### Objectives {7}

The primary objective of the study is to examine the effect of the Booster intervention on FSE and the other study outcomes: fatigue severity, school participation, life satisfaction, and perceived health. Study outcomes will be evaluated at the group level (research question (RQ) 1) in adolescents (12 to 18 years) with a chronic health condition in a stable phase. Participants will have experienced hindrance by fatigue for at least 3 months. We hypothesise that FSE increases first, followed after four weeks by decreased fatigue severity, and after six weeks by improved school participation, life satisfaction, and perceived health.

The secondary objective is to gain insight into individual differences regarding (moment of) significant change in outcomes during the Booster intervention. At the single-case level, we will explore which participants show change in outcomes (RQ2) and when these responders start to show change (RQ3). In addition, at group level, we will study whether baseline participant characteristics are predictive of improvements during the Booster intervention (RQ4) and whether long-term change can be observed (RQ5).

### Trial design {8}

This study employs a multiple baseline single-case experimental design (SCED). In a SCED, each case (i.e. each individual) serves as its own control, facilitated by intensive longitudinal data collection in two phases: Phase A (baseline) and Phase B (intervention) [36]. Randomisation of intervention start mitigates potential confounding of time due to external factors (*history*, e.g. transition from winter to summer*)* and internal factors (*maturation*, e.g. spontaneous recovery) [37]. This enhances internal validity [38], as does studying the effect across individuals with a multiple baseline design [38, 39]. Change relative to the randomised intervention start moments allows for causal inference within a SCED. Therefore, in this study, multiple participants start the Booster intervention at a randomised point in time to allow investigation of causal relationships between the intervention and study outcomes at the group level. Data is collected daily for 130 days (nearly nineteen weeks) with ESM via the Booster app on the participants’ phones.

**Fig. 1:**
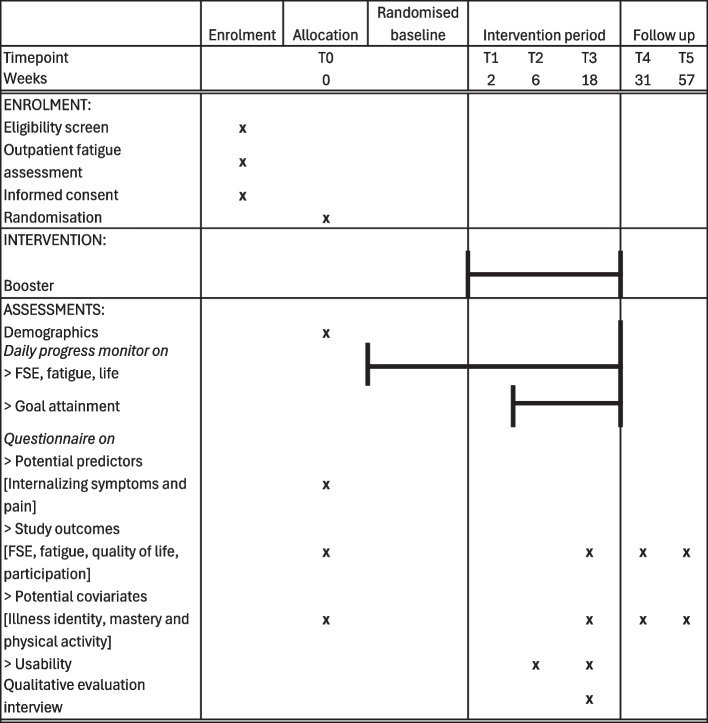
SPIRIT figure. Baseline and follow-up questionnaires for descriptives and covariates are filled out at T0 (baseline), T3, T4, and T5. T5 is one year after the Insight Conversation and the end of study participation. Abbreviation: FSE is fatigue-related self-efficacy

ESM is also known as ecological momentary assessment (EMA). ESM is a validated structured diary method integrated into daily life [40]. It captures personal experiences and behaviours along with real-time fluctuations in well-being through repeated surveys over a period ranging from several days to weeks [41]. In the following sections, the ESM use is detailed, both the daily ESM for measuring study outcomes in the daily progress monitor and the intensive ESM for personalised insight in fatigue. Based on the Internal Validity subscale of the 15-item Risk of Bias in *N*-of-1 Trials (RoBiNT) Scale, our study design has fair methodological rigour (Suppl. Table 1) [38, 42]. Examples of factors strengthening the internal validity are the long intensive longitudinal data collection and the measurement of intervention adherence. SPIRIT reporting guidelines were followed in this protocol [43]. An overview of the study design is given in Fig. 1 (Suppl. Fig. 1 for more details).

## Methods: participants, interventions, and outcomes

### Study setting {9}

Patients will be recruited from the Wilhelmina Children’s Hospital, University Medical Centre (UMC). We aim to include twenty participants. All participants have a chronic health condition (including but not limited to IBD or JIA) or a history of a life-threatening health condition (i.e. childhood cancer). Patients with a history of childhood cancer will be referred from the Prinses Maxima Centre for paediatric oncology to the specialist fatigue clinic at the Wilhelmina Children’s Hospital.

### Eligibility criteria {10}

Booster is intended for a heterogenous group of participants. Participants are eligible if they are at least 12 years and no older than 18 years at baseline; feel hindered by fatigue (≥ three months [44] with a paediatric short fatigue questionnaire (pSFQ) score ≥ 15 [45]); and are diagnosed with a chronic health condition or have been treated for childhood cancer. Participants will be excluded when another treatment for their fatigue than Booster is indicated or the Booster intervention is not feasible. The exclusion criteria concerning another indicated treatment are: a somatic or psychiatric diagnosis that fully explains the fatigue; a chronic health condition that has been unstable in the past three months (e.g. change in medication or cancer relapse); and significant functional limitations due to fatigue (e.g. school absenteeism > 50%). The exclusion criteria concerning feasibility are as follows: a cognitive impairment with an IQ < 70 as estimated by the treating physician; no possession of a smartphone with internet access; or unable to speak, read, understand or write Dutch.

As part of usual care, potential participants will undergo a standardised somatic and psychiatric fatigue analysis. Other exclusion criteria will be checked by the study team prior to enrolment.

#### Who will take informed consent? {26a}

Potential participants will receive oral and written information from the executive investigator.

#### Additional consent provisions for collection and use of participant data and biological specimens {26b}

Not applicable.

## Interventions

### Explanation for the choice of comparators {6b}

See section “Trial design”.

### Intervention description {11a}

#### Development

From September 2022 to March 2023, PROfeel was evaluated. This led to the development of a new intervention suited for early intervention (Suppl. Table 2a, b). In the iterative design process, based on Design Thinking [46], patients and healthcare professionals participated throughout (Suppl. Table 2c). The process started with interviews with former PROfeel users (i.e. (parents of) patients and healthcare professionals), followed by defining the development direction and ideation. Then, patients tested and provided feedback on consecutive app prototypes. An interdisciplinary team of game designers, medical doctors, and psychologists made design decisions. This resulted in the current intervention, renamed “Booster”. The new Booster app incorporates user preferences with features like a goal attainment module and motivating game mechanics as incentives (Suppl. Table 2d, Suppl. Figs. 2–5). The following sections describe the Booster intervention in detail.

#### Booster intervention

Booster is a patient-tailored intervention that aims to prevent the progression of fatigue and its impairments by increasing FSE. It blends on- and offline face-to-face contact with the executive investigator (hereafter: investigator), and mobile health (mHealth) support via the personalised Booster app (Suppl. Table 2d for an overview of app functionalities). Booster shifts the participant’s focus from fatigue to a modifiable area of daily life, further referred to as "lifestyle”. This shift is intended via increased insight into fatigue and related lifestyle factors through intensive ESM, followed by personalised goal-setting and -attainment (Suppl. Table 2b for Booster’s logic model). Booster comprises five stages: the Start Conversation, the Measurement Period, the Insight Conversation, the Experiment Period, and the Evaluation Conversation (Fig. 1, Suppl. Table 3), detailed in the following section. The daily progress monitor starts ten to eighteen days (depending on randomisation) before the Start Conversation. The participant will fill out this monitor via the Booster app throughout the intervention (see section “Plans for assessment and collection of outcomes”, Fig. 1 and Suppl. Fig. 1 and Suppl. Table 3).

##### Stage 1: Start Conversation

In a face-to-face meeting, the investigator explains the aim and the method of the Booster intervention to the participant, followed by information on the biopsychosocial model of fatigue (Suppl. Table 4 for an example biopsychosocial model). The participant and investigator discuss the participant’s attributes on fatigue-associated factors within the three domains. They focus on hypothesised inciting (e.g. disease flare) and sustaining factors (e.g. school pressure). This is the prelude to the personalisation of the ESM-items and -notification schedule by the participant in the Booster app. The participant is instructed to respond to ≥ 70% of the intensive ESM-surveys to provide sufficient measurement points to construct the Booster report.

**Fig. 2:**
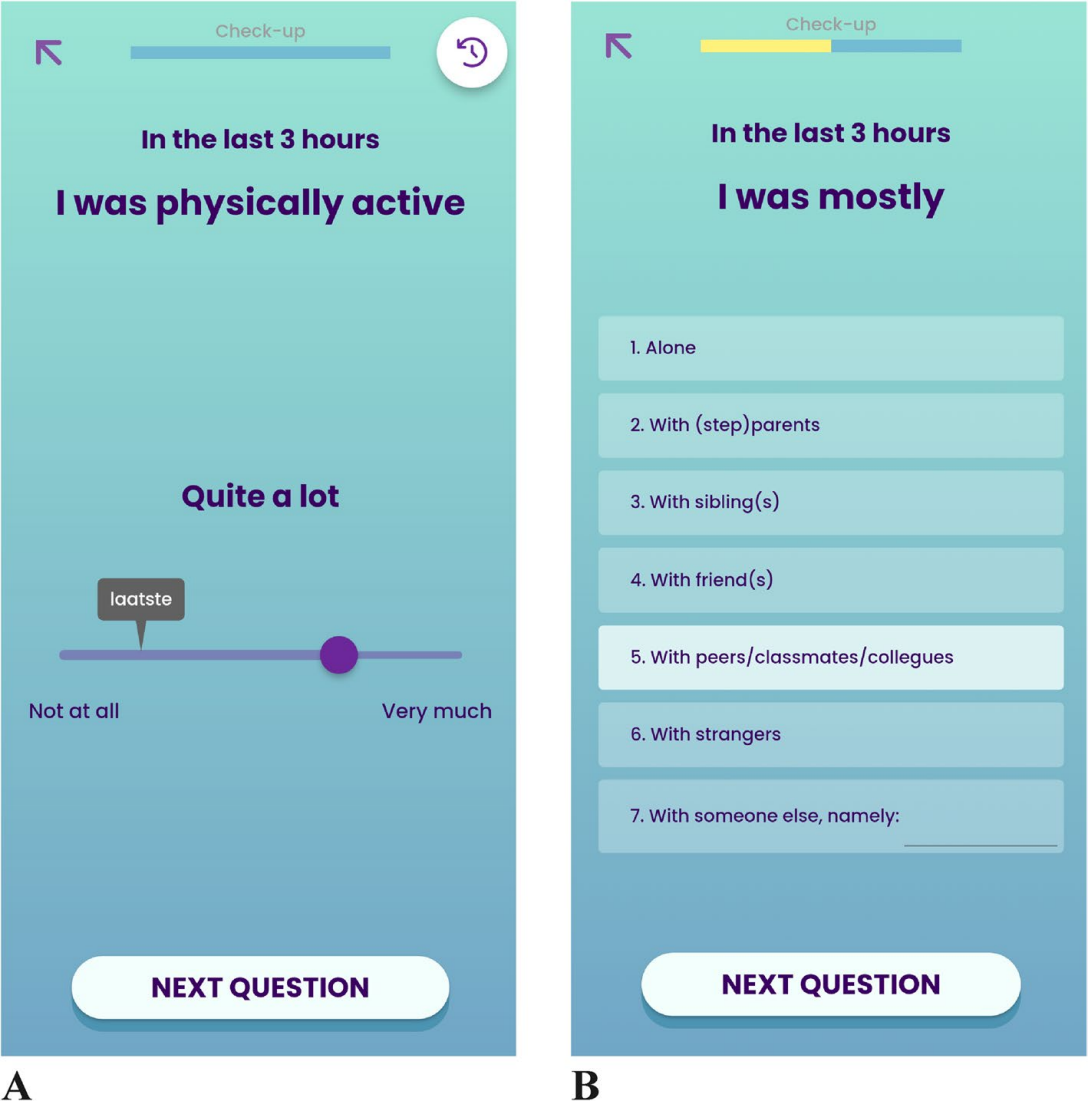
Examples of ESM-items for personalised insight in the Booster app. The survey is called “Check-up” All questions refer to the last three hours. **A** VAS-scale question, ranging from “Not at all” to “Very much”. The label “last” shows the response at the previous measurement time. The slider shows Likert scale-like options (“Quite a lot”). **B** Multiple choice question, displaying the standard (first six) options along with a personal option. The “someone else” options filled in previously will be shown in subsequent measurement times, with a maximum of two additional options

**Generic and personalised ESM-items for personalised insight** The intensive ESM-survey has generic and personalised items. Item domains are based on potentially fatigue-sustaining biological (e.g. sleep, physical activity), psychological (e.g. cognitions, feelings) and social (e.g. location and company of others) factors [22, 24]. Examples of generic items are as follows: “In the last 3 h, I felt fatigued” and “In the last 3 h, I was physically active”. For personalised items, the participant can choose the exact phrasing from a list or create their own phrasing (e.g. for positive affect, options such as “happy” or “enthusiastic” are available, Suppl. Table 5 for all ESM-items for personalised insight). Besides, the participant can add extra items, for example on specific physical symptoms [33, 34]. Most items are answered on a visual analogue scale (VAS) ranging from 0 (“not at all”) to 100 (“very much”). Likert options are shown on the answering scale (e.g. “a bit” for scores 21 to 40) instead of the exact score selected (e.g. 31). The rating from the previous measurement time will be visualised during the current measurement with the label “last”. This implicit reference point decreases measurement error and improves user experience [47]. For categorical items like location, the participant chooses between options or fills in the exact answer in the “other” option. This personalised option will be automatically added at the next measurement time (Fig. 2).

##### Stage 2: Measurement Period

For four weeks, the participant receives the intensive personalised ESM-survey five times daily via the Booster app with an approximate three-hour interval. The first survey of the day arrives at 9 AM, 10 AM, or 11 AM, based on the participant’s schedule choice during the Start Conversation. The exact timing of notification is randomly assigned within 30 min of the scheduled times. In case of non-response, reminders are sent after 20 and 60 min. After 90 min without response, the survey closes and is marked as missing. Completing the intensive ESM-survey costs less than one minute.

From PROfeel research [34], we know that a compliance of at least 35% (i.e. 49 completed surveys) is required to derive a Booster Report. Yet, the reliability of the output increases as the number of completed surveys does. Therefore, in line with previous research [48], we aim for at least 70% compliance. Extension of the Measurement Period will be offered to the participant in case of < 70% compliance. If the participant does not want to extend the Measurement Period, the researcher must discuss the content of the report with more caution because of the lower reliability (see section “Stage 3: Insight Conversation”).

##### Stage 3: Insight Conversation

After the four-week Measurement Period, the participant has a face-to-face conversation with the investigator. The Booster report, based on the intensive ESM-surveys, is discussed and lifestyle goals are set using shared-decision making.

**Fig. 3:**
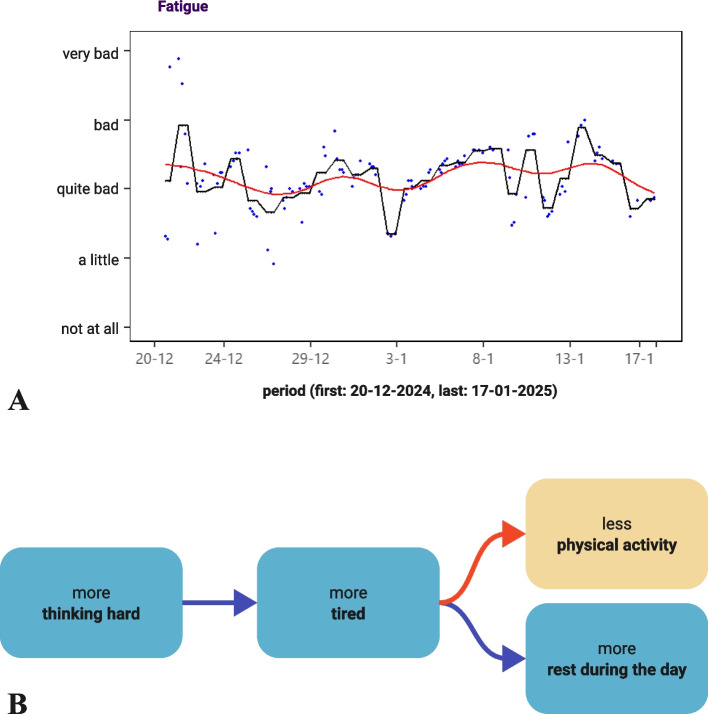
Example figures from Booster report. **A** Summary figure of fatigue intensity (y-axis) over the Measurement Period (28 days, x-axis). Blue dots are individual measurement points, black line is day average, red line is moving weekly average trend. **B** Dynamic network showing the time-lagged (3-h) relation between an increase in thinking hard first, followed by more fatigue, followed by less physical activity and more resting during the day

**Creating the Booster reportCreating the Booster report** A statistician, blinded to the participant, uses the intensive ESM surveys to compute an individual statistician report. This contains a summary of fatigue-sustaining factors and their dynamic networks. The summary includes fluctuations across the whole assessment period, averages for assessment hour and day of the week, differences for categorical factors, and day-to-day associations between paired ESM items across the Measurement Period. The dynamic networks (vector autoregression lag 1) are produced using Residual Dynamic Structural Equation Modelling. This method corrects for moving weekly average trends, weekend, and time of day and has been described more elaborately elsewhere [24, 48–50]. From all the generated output, the statistician and investigator independently select the most relevant summary figures and dynamic networks to be included in the Booster report (Fig. 3). Relevance is based on statistical significance, magnitude of standardised estimates, and assumed usefulness for the participant. Possible differences between selections are settled via discussion between the statistician and investigator.

**Discussion of the Booster report with the participant** During the Insight Conversation, the investigator discusses the Booster report with the participant. The investigator presents findings in the report as hypotheses. The participant is consistently prompted to share their interpretation and the recognisability of the findings (e.g. “Based on your measurements, it seems like…., do you recognise that?”).

**Fig. 4:**
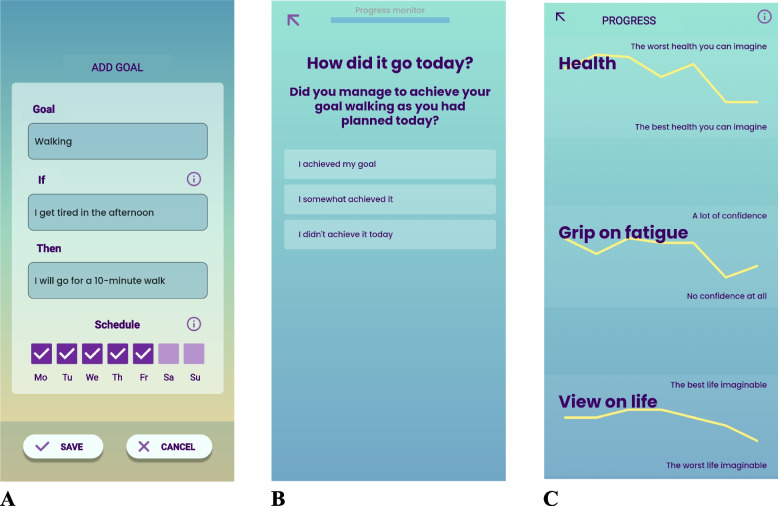
Booster’s Experiment Module. **A** Goal setting with implementation intention. A participant can add a goal by formulating what specific behaviour (“Then”) they aim to perform when (“If”). In the schedule, the days of the week are selected on which goal evaluation will take place. **B** Goal evaluation. In the daily progress monitor, the participant reflects on goal attainment on the goal days. **C** Progress tracking. Daily progress monitor outcomes can be tracked

**Shared decision-making on lifestyle goals** The Booster report discussion initiates the shared decision-making on personal lifestyle goals aimed at increasing FSE. The report serves as a clinical tool for goal-setting, though goals may also arise from other topics discussed during the conversation. Goals are tailored to the patient-specific needs. Guided by the investigator, the participant formulates personal lifestyle goals in the Booster app’s Experiment Module as an implementation intention (Fig. 4) [51]. Only one active goal is allowed at a time. Additionally, a larger overarching goal, or “Dream” (e.g. fully participating at school or having fun with friends), can be added to motivate progress on smaller goals.

##### Stage 4: Experiment Period

In the twelve-week Experiment Period, the Booster app aids goal attainment with evaluations added to the daily progress monitor on self-selected goal days. If the active goal is not reached multiple times, the app proposes to change or adapt the goal. The participant can adapt the active goal or change it to another goal.

##### Stage 5: Evaluation Conversation

The Booster intervention concludes with the Evaluation Conversation. Guided by the investigator, the participant reflects on insights gained, goals achieved, and effects noticed. Future steps for continuing with Booster insights are considered.

### Criteria for discontinuing or modifying allocated interventions {11b}

Due to the negligible risk of the intervention, no criteria for discontinuing or modifying it were specified. However, in case of a participant’s deteriorating health, the investigator may decide to withdraw the participant.

### Strategies to improve adherence to interventions {11c}

In the Measurement Period, to sustain motivation for completing ESM surveys, participants receive coins for each survey they fill out. Bonus coins are awarded for achievements such as completing a streak of five surveys in a row, maintaining good compliance, or reaching time-related milestones (e.g., being two weeks into the Measurement Period). Bonus points are accompanied by a badge, and all collected badges can be viewed in the app. Points can be used in the in-app game. In the game, the participant pilots the avatar over an increasingly fast side-scrolling track by tapping the screen to avoid obstacles. In the game store, the participant can use the points to buy new track elements to create, for example, a longer, easier or prettier track. Additionally, the participant can create a daily diary post by selecting a sticker and/or writing things down. This results in a personal overview (Suppl. Table 2 d, Suppl. Fig. 3c).

In the Experiment Period, on the home screen, the app shows the active goal and its attainment. Besides, the Booster report becomes visible in the app. Also, the participant can track their own daily progress monitor outcomes.

Throughout the study period, participants will receive weekly reminders that they may contact the investigator for support. If a decline in adherence is detected, motivational messages will be delivered via the Booster app to encourage continued engagement.

### Relevant concomitant care permitted or prohibited during the trial {11d}

Participants are ineligible if they are already involved in a behavioural intervention targeting fatigue. However, they may receive concomitant fatigue-focused care if it is part of the patient-tailored Booster lifestyle goals. Participants may also continue with any medical or psychological care that began before the Booster intervention, provided the primary focus of this treatment is not on reducing fatigue.

### Provisions for post-trial care {30}

At the end of the Evaluation Conversation, the investigator identifies any ongoing care needs not addressed by the Booster intervention and ensures appropriate referrals are made.

### Outcomes {12}

The primary outcome of the study is FSE. Other outcomes are fatigue severity, school participation, life satisfaction, and perceived health (see “Plans for assessment and collection of outcomes”).

### Participant timeline {13}

Baseline assessment (T0) will take place ten to eighteen days before the Start Conversation (T1, Fig. 1). In this interim period, the participant starts filling out the daily progress monitor via the Booster app, which continues until the Evaluation Conversation (T3). From the start of the Experiment Period onwards (T2 to T3), marked by the Insight Conversation, goal attainment is evaluated as well via the Booster app (see sections “Stage 4: Experiment Period” and “Goal attainment”). Follow-up with elaborate questionnaires (see section “Demographics and potential covariates”, Suppl. Table 6) takes place three, six, and twelve months after the Insight Conversation (T2). Baseline and follow-up questionnaires will be filled out in the web-based survey portal KLIK (www.hetklikt.nu) (Fig. 1 and Suppl. Table 3).

### Sample size {14}

The power to detect an intervention effect with a multiple baseline design (RQ1) depends on the autocorrelation of consecutive daily progress monitor measurements, the start moment range, the number of repeated measurements, the intervention effect, and the number of cases [52]. In the earlier PROfeel study [48], the median autocorrelation of weekly measurements was 0.17 for FSE, 0.30 for fatigue severity, and 0.09 for QoL. Median autocorrelation of the five times daily ESM item on fatigue severity was 0.37 [48]. We expect the daily measurements in this study to show a higher autocorrelation than weekly measurements, but lower than a five times daily item. Thus, we assumed the autocorrelation in our study to be 0.30 across outcomes. The start moment range is nine, as the Booster intervention is randomised to start (T1) ten to eighteen days after the start of the daily progress monitor (T0). With 100% compliance, the number of repeated measurements is 130. With the abovementioned assumptions and inclusion of twenty cases, with up to 30% of daily measurements missing, the multiple baseline single-case randomisation test (SCRT) can detect medium to large intervention effects of ≥ 0.7 with a power of ≥ 0.80 at the group level (RQ1, Table 1). However, we anticipate small intervention effects since Booster is an early intervention, not treatment. The SCRT, used in SCED, has limited power to detect such small intervention effects [37, 52, 53]. In contrast, the permutation distancing test (PTD), developed for testing differences in means in single-case observational designs (SCODs), exhibits higher statistical power [53]. It is important to note that SCOD cannot establish causality. With the abovementioned assumptions, with up to 40% of daily measurements missing, the study is well powered to observe changes in outcomes for effect sizes as small as 0.3 at the single-case observational level (RQ2, Table 1).

**Table 1:** Power for detecting intervention effects at the group and single-case level

Factors influencing power	Study design	
Autocorrelation (%)	30	
Repeated measurements (*n*)	130	
Power (%)	≥ 80	
Cases (*n*)	20 (group level)	1 (single-case)
Test	Multiple baseline SCRT	PDT
Start moment range (*n*)	9	NA
Maximum percentage missing data to retain sufficient power (%)	30	40
Minimum detectable effect size	0.7 (medium to large)	0.3 (small)

At the single-case level, intervention effect sizes are defined as small if 0.00–0.99, as medium if 1.00–2.49, andas large if ≥ 2.50 [54]

*Abbreviations: NA* is not applicable, *PDT* is permutation distancing test, *SCRT* is single-case randomisation test

For 80% power in detecting change points (RQ3), fifty measurements are needed, including at least six post-change points (start Phase B) [55]. For every outcome, the number of Phase A measurements is at least 42 (six weeks) if 100% compliant (see section “Statistical methods”). For detecting change points specifically, change points up until six days before the end of the daily progress monitor can be detected. The start of the daily progress monitor ten to eighteen days before the Start Conversation (T1) enables us to detect whether the Start Conversation itself or the Measurement Period (T1 to T2) coincides with a change point (Suppl. Figure 1).

### Recruitment {15}

Participants meeting the inclusion criteria will be approached by their treating paediatrician.

### Assignment of interventions: allocation

#### Sequence generation {16a}

A randomisation sequence will be generated in advance using a computer algorithm. Each value in the sequence corresponds to a number of days between ten and eighteen, determining the timing of the intervention period (T1) relative to the start of the daily progress monitor (T0).

#### Concealment mechanism {16b}

The pre-generated randomisation sequence will be stored in a fixed list. Participants will be assigned start dates based on the order of recruitment, following the sequence exactly. This procedure ensures consistent application of random allocation while avoiding selection bias.

#### Implementation {16c}

Investigators enrolling participants will assign each individual the next start date in the sequence. If a participant’s assigned start date falls on a weekend, the intervention will begin on the nearest weekday (the preceding Friday or following Monday).

### Assignment of interventions: blinding

#### Who will be blinded {17a}

The analysis of the ESM data will be performed by an independent statistician with no involvement in participant recruitment or access to identifying information. Both the statistician and the investigator will refer to participants solely by their study codes (e.g. B02) to ensure confidentiality and minimise bias.

#### Procedure for unblinding if needed {17b}

Not applicable.

### Data collection and management

#### Plans for assessment and collection of outcomes {18a}

Study outcomes will be assessed using the daily progress monitor, a repeated survey. The Booster app sends the daily progress monitor at a random time within the hour before bedtime. Participants determine the exact timing of completing the monitor, provided it is within three hours of receiving the notification. Responses not submitted within this three-hour window are considered missing. For each item, participants are asked to reflect on their day. Item order is randomised to minimise method bias [56]. The daily monitoring period starts ten to eighteen days before the Start Conversation and continues for 130 days, ending twelve weeks into the Experiment Period.

#### Fatigue-related self-efficacy

To make the daily progress monitor feasible, a single item for FSE was constructed to measure one’s confidence in dealing with fatigue. Face validity was confirmed after three rounds of interviews with adolescents with (a history of) severe fatigue. The items tested in each round were based on items from validated self-efficacy questionnaires (Suppl. File 1). The resulting item (translated from Dutch) is “Today I believed that I could prevent fatigue from bothering me”. FSE will be scored on a VAS scale from 0 (“I didn’t believe it at all”) to 100 (“I believed it completely”), with higher scores indicating higher FSE. Likert options are shown on the answering scale (e.g. “I believed it a bit” for scores 21 to 40) instead of the exact score selected (e.g. 26).

#### Other study outcomes

*Fatigue severity* will be measured with the four-item pSFQ, answered on a seven-point Likert scale from “Yes, that is correct” to “No, that is not correct” [45]. The pSFQ score ranges from 4 to 28, with higher scores reflecting more severe fatigue. It has good psychometric properties, including Cronbach alpha’s ranging from 0.84 to 0.94 [45]. In the official pSFQ, respondents are instructed to think of the past two weeks. For the daily progress monitor in this study, the instruction was adapted to think of today.

*Participation* will be measured as school or work presence, which is calculated by the hours present divided by the hours scheduled multiplied by 100. For each working or school day, participants will fill in the number of hours on their personal timetable and the number of hours attended. This item is adapted from the PROactive cohort study [57].

*Life satisfaction* will be measured with the Cantril Ladder, adapted for adolescents [58]. Respondents rate their life with a ladder numbered from zero to ten. The bottom represents “The worst possible life”, and the top ‘The best possible life”. This version of the Cantril Ladder is extensively used in the Netherlands. It has good reliability and convergent validity with other well-being measures [58].

*Perceived health* will be measured with the EuroQol (EQ) visual analogue scale (VAS) [59]. Respondents rate their current health on a vertical scale from 0 (“The worst health you can imagine”) to 100 (“The best health you can imagine”). The scale shows the exact number selected. Rating their health status is feasible for adolescents, and EQ-VAS scores can predict health-related QoL as measured by the EQ Five Dimensions Health Questionnaire (EQ-5D) [59].

#### Goal attainment

The daily progress monitor will also be used to evaluate goal attainment on a personalised frequency during the Experiment Period (see section “Stage 4: Experiment Period”). For example, if the goal is to cycle to school on Mondays, Thursdays, and Fridays, the goal evaluation will only take place on those days.

#### Demographics and potential covariates

To characterise participants, the following parameters will be obtained at baseline: age, sex, primary diagnosis and time since diagnosis, fatigue duration, and level of education. Additional questionnaires to be completed at baseline and follow-up are the multiple-item questionnaires measuring the same constructs as measured by the daily ESM progress monitor. Moreover, potential predictors, mediators, and moderators of the primary outcome FSE will be assessed. These potential covariates are internalising symptoms, pain, illness identity, mastery, and physical activity (Suppl. Table 6 for an overview of baseline and follow-up assessments).

##### Qualitative evaluation

A semi-structured interview will be conducted with each participant at the Evaluation Conversation by the investigator. The first aim is to gain understanding of Booster’s effect, and internal and external factors influencing this effect, as experienced by the participant. The second aim is to evaluate the Booster intervention in general and the Booster app specifically. Before the interview, participants will fill in a short Booster-tailored usability questionnaire (Suppl. File 2). The questionnaire will also ask about the effect of Booster (e.g., “What is the effect of Booster on you?”). In the second part of the interview, follow-up questions will be asked about motivation, feasibility, and usability (Suppl. File 3). This will guide potential future development steps. Interviews will be audio recorded and transcribed verbatim. The interview and the usability questionnaire combined will support the interpretation of the quantitative single-case outcomes.

#### Plans to promote participant retention and complete follow-up {18b}

To reduce the burden of repeatedly filling out the daily progress monitor, we aimed to include as few items as possible for each construct while maintaining good psychometric properties. To increase compliance, game mechanics were added (see section “Stage 2: Measurement Period”) and the daily assessment time is personalised based on participants’ bedtimes.

#### Data management {19}

A data management plan has been written and reviewed by UMC Utrecht’s data quality management team.

#### Confidentiality {27}

All data will be handled in accordance with the General Data Protection Regulation (GDPR). Only members of the research team directly involved in the study will have access to participant codes and associated data. Electronic data will be stored on secure, password-protected systems that comply with UMC Utrecht’s information security standards. Physical documents, including signed informed consent forms, will be stored in a locked cabinet at the Wilhelmina Children’s Hospital. In the event of data sharing after the study has ended, all datasets will be anonymised to protect participant privacy prior to release.

#### Plans for collection, laboratory evaluation and storage of biological specimens for genetic or molecular analysis in this trial/future use {33}

Not applicable.

## Statistical methods

### Statistical methods for primary and secondary outcomes {20a}

#### RQ1: What is the effect of the Booster intervention on the study outcomes at the group level?

We will study change in mean level of outcomes (level change) after outcome-specific lags (Suppl. Figure 1). We hypothesise that FSE, Booster’s main target, will improve immediately after the Insight Conversation. Improvements in fatigue and participation are expected four weeks later, with life satisfaction and perceived health improving at six weeks post-Insight Conversation. After visual inspection of the data, the multiple baseline SCRT will determine the presence of significant medium to large Booster intervention effects at the group level. The null hypothesis is that observed outcomes are independent of intervention phase. The randomisation of intervention start is a precondition for conducting the SCRT. It tests significance by comparing the observed test statistic (mean level change) to the test statistic distribution of all other possible intervention start points. This controls for time-related confounding [60, 61]. The SCRT outcomes will show if the Booster intervention is effective at the group level.

#### RQ2: What changes are observed in study outcomes at the single-case level during the Booster intervention?

Second, we will use the permutation distancing test (PDT) and single-case interrupted time series analysis (ITSA) to explore changes at the single-case level during the Booster intervention. The PDT will test the null hypothesis that observations within one individual are evenly distributed between Phase A and B. The PDT compares the observed test statistic (mean level change) to the test statistic distribution of permuted orders of observations. To create this distribution, the PDT uses different subsets of observations with a specific time distance to compensate for serial autocorrelation [53]. With the ITSA, a regression model is fitted to the data, allowing for separate intercepts and slopes in Phase A and B, while correcting for serial autocorrelation [54]. Although neither test can directly establish causality (e.g. the PDT was developed specifically for AB-phase SCODs) [53], together they complement the group-level SCRT results by identifying which individuals show significant change in outcomes in Phase B (responders) and which do not (non-responders). This triangulation approach leverages the strengths of both tests (e.g. the non-parametric PDT has no distributional assumptions, the ITSA enables trend analysis) and provides a comprehensive understanding of the data. In particular, the ITSA helps determine whether significant level changes found could be attributed to trends in the data instead of a possible intervention effect [54]. The combined results from both statistical tests strengthen the overall evidential basis, suggesting increased robustness and reliability of the findings.

#### RQ3: What is the pattern of change in the study outcomes at the single-case level?

We will conduct single-case change point analyses for participants in which significant changes in mean level or slope were detected by the PDT and/or ITSA (RQ2). Data will be analysed with Wild Binary Segmentation (WBS) [62]. WBS has no assumptions. It tests for change points by splitting the longitudinal observations into random intervals. Per individual, the analysis gives insight into the exact moment of change (both level and slope) for each outcome. This will provide us with information on which Booster intervention stages or elements were relevant to changes in the study outcomes across individuals. In the future, this information on effective intervention mechanisms could be used to improve the Booster intervention further.

#### RQ4: Which characteristics are predictive of changes in the study outcomes during the Booster intervention?

To explore participants’ characteristics that may predict changes in outcomes (see section “Objectives”), multiple analyses are planned. First, a multi-level analysis will be used to study the relation between self-reported goal attainment and changes in outcomes. Second, participants will be grouped into responders and non-responders based on a significant difference in FSE with the PDT (RQ2). We will describe and compare the (baseline) characteristics of responders versus non-responders (e.g. baseline FSE and fatigue, duration of symptoms, school absence, and illness identity), using the Mann–Whitney *U* test for continuous predictors, and a Fisher’s Exact Test for categorical data.

#### RQ5: What long-term change in outcomes is observed at the group level?

Baseline (T0) and follow-up questionnaires (T3 (end of intervention), T4 (3 months after T3), T5 (6 months after T4) will be used to assess long-term change in measured outcomes (i.e. FSE, fatigue, participation, life satisfaction, and perceived health), moderators (i.e. illness identity and mastery) and mediators (i.e. physical activity and sleep) at group level (Fig. 1). We will use linear mixed-effects models with time as a fixed effect and a random intercept for participants to account for the repeated follow-up measurements within individuals. If model assumptions are severely violated, a non-parametric alternative will be considered.

### Interim analyses {21b}

Not applicable.

### Methods for additional analyses (e.g. subgroup analyses) {20b}

Not applicable.

### Methods in analysis to handle protocol non-adherence and any statistical methods to handle missing data {20c}

If missing data exceeds 30–40%, we consider imputation where possible to retain power (Table 1). To guide this decision, we will first examine the missing data mechanism to determine if imputation is valid and, if so, which method is most suitable, such as using multiple imputation methods suitable for time-series data [63].

### Plans to give access to the full protocol, participant level-data and statistical code {31c}

External researchers can apply for access to the data by submitting their research proposal to the principal investigator of this study. Studies concerning youth with chronic illnesses, fatigue, and those involving extensive repeated measurements may be eligible to use the data.

## Oversight and monitoring

### Composition of the coordinating centre and trial steering committee {5d}

The Wilhelmina Children’s Hospital, part of the UMC Utrecht, is the coordinating centre of the present study. The research group, including the principal and executive investigators, will meet weekly to manage the study.

### Composition of the data monitoring committee, its role and reporting structure {21a}

As the study is not subject to the Medical Research Involving Human Subjects Act, no data monitoring committee has been created.

### Adverse event reporting and harms {22}

No study-related adverse events are expected due to this negligible risk. In case of adverse events, these will be documented, and necessary medical follow-up will be provided.

### Frequency and plans for auditing trial conduct {23}

As the study is not subject to the Medical Research Involving Human Subjects Act, no independent monitor is appointed.

### Plans for communicating important protocol amendments to relevant parties (e.g. trial participants, ethical committees) {25}

Modifications will be shared via the website of the eHealth Junior Consortium (ehealthjunior.nl) after consultation of the research quality coordinator of the UMC Utrecht.

### Dissemination plans {31a}

We intend to share the results of the described study with the scientific community via (inter)national conferences and publication(s) in peer-reviewed journal(s). Results will be shared with a wider public via eHealth Junior Consortium partners, including patient organisations, as well as via popular science articles.

## Discussion

Fatigue is a common debilitating symptom in children with a chronic health condition. At an early stage, the personalised transdiagnostic intervention Booster could prevent persistent fatigue and its impairments by increasing FSE. It makes use of innovative methods by combining tailored ESM-insight, mHealth and healthcare professional support. This single-case multiple baseline study examines Booster’s effect on FSE and other outcomes. Understanding of the group level results will be enhanced by extensive explorative analyses at the single-case level. Furthermore, this study enables a deeper understanding of participant characteristics related to change.

A key strength is exploring ESM’s potential as a clinical tool in paediatric health care, as a step towards its implementation. The use of ESM for personalised insight is non-existent in paediatric clinical practice [64, 65]. Neither has ESM as an intervention been studied in children with somatic health conditions [64], except in earlier PROfeel studies [33, 34, 48, 50]. Other studies did show ESM’s value in adolescent [66, 67] and adult mental health care [68–70], and even specifically for fatigue after cancer-treatment [71, 72]. This study aims to pioneer the use of ESM for personalised insight in paediatric health care, building on its established success in adolescent and adult mental health.

Second, building on PROfeel’s feasibility and usefulness for children with chronic health conditions [50], and its effectiveness in youth with persistent fatigue [33, 34], this study addresses three major remaining questions: (1) is ESM for personalised insight effective as early intervention? (2) which intervention elements influence which outcomes for whom, and when? and (3) how do adolescents evaluate the Booster intervention in general and the app specifically?

With regard to (1), the multiple baseline SCED enables showing effectiveness at the group level, while limiting the sample size needed compared to a traditional randomised controlled trial, saving time and resources.

With regard to (2), the intensive longitudinal data collection in the single-case design will clarify the preventative working mechanisms of a paediatric ESM intervention. Although we hypothesise that FSE change coincides with the Insight Conversation, change might already occur during the Measurement Period. Filling out ESM surveys may be an intervention on its own, as self-monitoring can increase emotional awareness and reduce symptoms of depression in young people [66, 67]. Collecting data on multiple outcomes before and during each Booster stage, followed by the change-point analyses (RQ3), will give direction on which intervention elements affect specific outcomes for whom.

With regard to (3), Booster is the result of an extensive iterative and multistakeholder codesign process. The largest changes were made to the smartphone app, in which game mechanics (e.g. rewards and a minigame) were incorporated to sustain motivation throughout the intervention, and the Experiment Module was added. Involving adolescents in the design process ensured a greater understanding of their needs and gave room to incorporate their ideas. This could increase the success of the intervention [73]. A strength of this study is the ongoing input from adolescents. Through the usability questionnaire and qualitative evaluation, their feedback will inform and guide future developments.

A third strength of this study is the embedding in the interdisciplinary eHealth Junior consortium (ehealthjunior.nl) with the involvement of researchers with diverse expertise (e.g. ESM-methodology, psychology, paediatrics, implementation sciences, and game design) and non-academic partners (e.g. patient associations and health care insurers) is secured [74].

However, the study faces several challenges. First, in order to mirror the heterogeneous clinical practice, the inclusion criteria are broadly defined, with a low fatigue severity threshold of ≥ 15 points with the pSFQ. This corresponds to the upper 50th percentile of children with a chronic health condition) [45]. This may result in a diverse group of participants, limiting result replication. Nevertheless, this approach acknowledges the large intra-individual differences in fatigue and thereby strengthens the ecological validity of our results. Also, the additional analyses at the single-case level will shed light on intra-individual differences (RQ2 and 3). Ultimately, our study aims to identify the value of the Booster intervention for adolescents with a chronic health condition who experience hindrance by fatigue.

Second, the study is only powered to demonstrate causal medium to large intervention effects at the group level (RQ1),[37, 52] while one might expect smaller effects. Nevertheless, these smaller intervention effects can be observed with the PDT at the single-case level (RQ2), albeit without causal inference. However, if effects are replicated across individuals and also align with the timing of specific Booster intervention stages, the results of the PDT build a case for treatment efficacy, making alternative causal explanations less likely [39].

Third, we must consider the potential detrimental effect of intensive longitudinal data collection. While most participants in ESM studies reported heightened awareness from repeated measurements as positive, some experienced adverse effects [50, 75–77]. Repeated self-reflection can lead to effective cognitive processing and problem-solving but may also increase internalising symptoms [78]. In our study, intensive longitudinal data collection occurs through ESM surveys during the four-week Measurement Period and the daily progress monitor throughout. It serves both treatment and research purposes. We carefully balanced measurement burden (e.g. minimising items, personalised timing, distraction options in the Booster app, and contact with the investigator, exploring long-term effects with pre-post design instead of extending the daily progress monitor) with validity and scientific rigour (e.g. personalised Booster reports, single-case insights, validated items with strong psychometric properties, and sufficient measurements for statistical power).

A fourth limitation of this study is that to minimise the measurement burden, our primary outcome is measured with a newly developed single-item question, which risks the generalisability of the findings. To minimise this, in the development of the item, we followed the guide by Bandura to construct self-efficacy scales [79], and established face validity with the target population. In the analyses, we will check concurrent validity via correlation with other outcome measures and the baseline and follow-up questionnaires [80]. Afterwards, we will add the item to the ESM-item repository (esmitemrepository.com) for future use by other researchers.

To conclude, we will study the effect of the Booster intervention at the group level while also providing a more detailed understanding at the single-case level by using a multiple baseline SCED. This study builds on earlier studies with PROfeel, Booster’s predecessor. The intervention has evolved in collaboration with patients, clinicians and interdisciplinary stakeholders from the eHealth Junior consortium. Booster aims to avert progression of fatigue and to prevent its impairments in children with chronic health conditions by increasing FSE. It is an example of an in-depth investigation of an early intervention for which small intervention effects are expected. This study will be a stepping stone for the implementation of ESM tools for personal insight in clinical practice.

### Trial status

This is the first version of the protocol (March 4 2025). Recruitment started in October 2024. End of recruitment is anticipated in April 2025.

## Supplementary Information

Supplementary Material 1: Supplementary Table 1. Risk of Bias in *N*-of-1 Trials (RoBiNT) scale for Booster study 2. Supplementary Fig. 1. Study overview 4. Supplementary Table 2a. Booster’s guiding principles 5. Supplementary Table 2b. Booster’s logic model 6. Supplementary Table 2c. Participatory design process 9. Supplementary Table 2d. Booster app functionalities 10. Supplementary Fig. 2. The game 11. Supplementary Fig. 3. Other general functionalities 12. Supplementary Fig. 4. Measurement Period-specific functionalities 13. Supplementary Table 3. Participant timeline 14. Supplementary Table 4. Example of a biopsychosocial model of fatigue 15. Supplementary Table 5. Items in ESM-survey of Measurement Period 16. Supplementary Table 6. Proposed covariates measured at baseline and follow-up 17. Supplementary File 1. Construction and validation of a single Item for measuring fatigue-related self-efficacy 18. Supplementary File 2. Usability questionnaire 19. Supplementary File 3. Semi-structured interview – topic guide 20. Supplementary references.

## Data Availability

Data will be available after publication and for at least one year after the end of the eHealth Junior Consortium. Data will remain available until the end of 2040. For more information, see section “Confidentiality”.

## Abbreviations

EMA: Ecological momentary assessment
EQ: EuroQol
EQ-5D: EQ Five Dimensions Health Questionnaire
ESM: Experience sampling methodology
FSE: Fatigue-related self-efficacy
IBD: Inflammatory bowel disease
ITSA: Interrupted time series analysis
JIA: Juvenile idiopathic arthritis
mHealth: Mobile health
PDT: Permutation distancing test
QoL: Quality of life
RQ: Research question
RoBiNT: Risk of Bias in N-of-1 Trials Scale
SCED: Single-case experimental design
SCOD: Single-case observational design
SCRT: Single-Case Randomisation Test
UMC: University medical centre
VAS: Visual analogue scale
WBS: Wild Binary Segmentation
