# Supplementary material for: Blended therapy for adolescents with chronic health conditions to increase fatigue-related self-efficacy (Booster): protocol for a single-case multiple baseline study
Source: Trials. 2025 Jul 24;26:255. doi: 10.1186/s13063-025-08960-1 (PMC12291284; doi:10.1186/s13063-025-08960-1)
Supplement: Supplementary file 1 — Supplementary Material 1: Supplementary Table 1. Risk of Bias in N-of-1 Trials (RoBiNT) scale for Booster study 2. Supplementary Fig. 1. Study overview 4. Supplementary Table 2a. Booster’s guiding principles 5. Supplementary Table 2b. Booster’s logic model 6. Supplementary Table 2c. Participatory design process 9. Supplementary Table 2d. Booster app functionalities 10. Supplementary Fig. 2. The game 11. Supplementary Fig. 3. Other general functionalities 12. Supplementary Fig. 4. Measurement Period-specific functionalities 13. Supplementary Table 3. Participant timeline 14. Supplementary Table 4. Example of a biopsychosocial model of fatigue 15. Supplementary Table 5. Items in ESM-survey of Measurement Period 16. Supplementary Table 6. Proposed covariates measured at baseline and follow-up 17. Supplementary File 1. Construction and validation of a single Item for measuring fatigue-related self-efficacy 18. Supplementary File 2. Usability questionnaire 19. Supplementary File 3. Semi-structured interview – topic guide 20. Supplementary references. [file 13063_2025_8960_MOESM1_ESM.docx]

**Supplementary Material**

[Supplementary Table 1. Risk of Bias in *N*-of-1 Trials (RoBiNT) Scale for Booster Study 2](#_Toc199511076)

[Supplementary Figure 1. Study overview 4](#_Toc199511077)

[Supplementary Tables 2a-d. The Development and Design of the Booster Intervention 5](#_Toc199511078)

[Supplementary Table 2a. Booster’s Guiding Principles 5](#_Toc199511079)

[Supplementary Table 2b. Booster’s Logic Model 6](#_Toc199511080)

[Supplementary Table 2c. Participatory Design Process 9](#_Toc199511081)

[Supplementary Table 2d. Booster App Functionalities 12](#_Toc199511082)

[Supplementary Figure 2. The game. 16](#_Toc199511083)

[Supplementary Figure 3. Other general functionalities. 17](#_Toc199511084)

[Supplementary Figure 4. Measurement Period-specific functionalities. 18](#_Toc199511085)

[Supplementary Table 3. Participant Timeline 20](#_Toc199511086)

[Supplementary Table 4. Example of a Biopsychosocial Model of Fatigue 21](#_Toc199511087)

[Supplementary Table 5. Items in ESM-survey of Measurement Period 21](#_Toc199511088)

[Supplementary Table 6. Proposed Covariates Measured at Baseline and Follow-up 23](#_Toc199511089)

[Supplementary File 1. Construction and Validation of a Single Item for Measuring Fatigue-related Self-efficacy 24](#_Toc199511090)

[Supplementary File 2. Usability Questionnaire 26](#_Toc199511091)

[Supplementary File 3. Semi-structured Interview – topic guide 28](#_Toc199511092)

[Supplementary References 29](#_Toc199511093)

## Supplementary Table 1. Risk of Bias in *N*-of-1 Trials (RoBiNT) Scale for Booster Study

The Booster study design scored 22 out of 30 points, with 8/14 points of the Internal Validity subscale and 14/16 points of the External Validity subscale.

| **Internal validity** | | |
| --- | --- | --- |
| Item | RoBiNT Scale | Details (points) |
| 1 | Design | A nonconcurrent multiple baseline design of twenty cases divided over two tiers of nine possible start moments. (1) |
| 2 | Randomisation | The start of the Booster intervention (T1) is randomised after ten to eighteen days of filling out the daily ESM progress monitor (T0) (i.e., nine randomisation options). The randomisation results in different Phase A lengths across participants. For the primary outcome FSE, Phase A will therefore last between 38 and 46 days. (2) |
| 3 | Sampling behaviour (all phases) | Phase A and B will have a minimum duration of 56 days for study outcomes. Daily sampling will take place via the daily ESM progress monitor. Even with low compliance, the number of measurement points will exceed five. (2) |
| 4 | Blinding patient/therapist | Blinding of the participant and the investigator for phase is not possible for the investigator guides the patient through the intervention. (0) |
| 5 | Blinding assessors | The participant acts as the assessor by self-reporting on outcomes via the daily ESM progress monitor. The participant is independent of the investigator but is not blinded for phase. (1) |
| 6 | Inter-rater reliability | All outcomes are measured by self-report via the daily ESM progression monitor. (0) |
| 7 | Treatment adherence | Adherence to experimenting with goal attainment is defined as filling in the daily ESM progress monitor during the Experiment Phase via the Booster app. Log data for all survey responses is automatically saved. (2) |

| **External validity and Interpretation** | | |
| --- | --- | --- |
| **Item** | **RoBiNT Scale** | **Details (points)** |
| 8 | Baseline characteristics | Baseline characteristics will be obtained via the baseline self-report questionnaires. They include age, sex, aetiology and severity of fatigue. Also, qualitative evaluation of the patient’s motivation and expectancies for Booster will take place at intake by the investigator. (2) |
| 9 | Setting | The face-to-face Booster conversations (stages 1, 3 and 5) will take place in the hospital or with video calling, possibly joined by parent(s). The other intervention elements are intertwined with daily life of participants (e.g., at home, school, work or friend’s), for which no precise description is possible. (1) |
| 10 | Dependent variable (target behaviour) | Target behaviour will be measured via the FSE-item in the daily ESM progress monitor. (2) |
| 11 | Independent variable (intervention) | The Booster intervention is described in detail in section 2.4.2 Booster Intervention, including content and procedural details. (2) |
| 12 | Raw data record | Twenty cases will be conducted. Raw data (i.e., all samplings of the daily ESM progress monitor), will be presented for three cases. (2) |
| 13 | Data analysis | Data will primarily be analysed with the SCRT at the group level. The statistical analysis is described in detail in section 2.7 Intended Statistical Analysis. (2) |
| 14 | Replication | With twenty patients, the case will be replicated nineteen times. (2) |
| 15 | Generalisation | Participants will fill out validated questionnaires at baseline (pre-treatment) and follow-up (post-treatment). (1) |

Abbreviations: FSE is fatigue-related self-efficacy, SCRT is single case randomisation test.


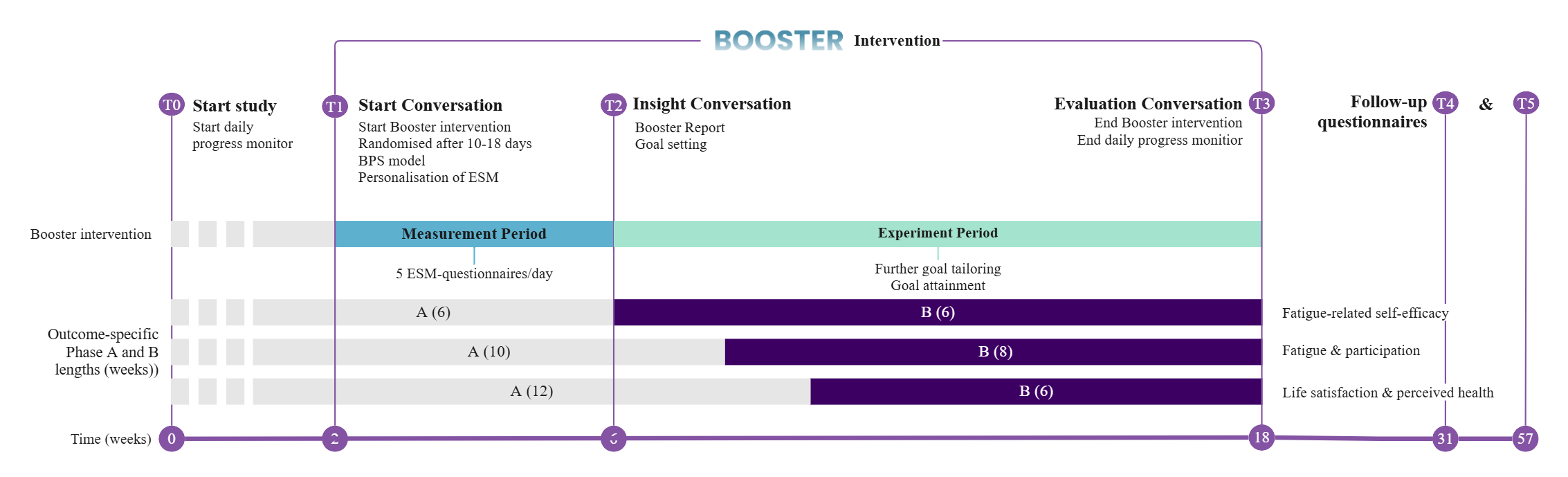


## Supplementary Figure 1. Study overview

Study timeline (T0 to T5) is shown, relative to Booster intervention timing and outcome-specific Phase A and B lengths. The start of Booster intervention is randomised to 10 – 18 days after start of daily progress monitor. Baseline and follow-up questionnaires for descriptives and covariates are filled out at T0 (baseline), T3, T4 and T5. T5 is one year after the Insight Conversation and the end of study participation. Abbreviations: A is Phase A (baseline), B is Phase B (intervention), BPS is biopsychosocial model, ESM is experience sampling method.

## Supplementary Tables 2a-d. The Development and Design of the Booster Intervention

The Booster intervention is the result of a participatory iterative design process that followed the completion and evaluation of the PROfeel intervention in a randomised crossover trial.^2–4^ The following tables give further information on the intervention. Suppl. Table 2a describes the overarching guiding principles for the intervention. Suppl. Table 2b shows Booster’s logic model, an overview of the key components of the intervention and working mechanisms. The major changes from PROfeel to Booster are highlighted. Suppl. Table 2c describes the participatory design process. Suppl. Table 2d details the Booster app functionalities and their rationales.

### Supplementary Table 2a. Booster’s Guiding Principles

| **User Context** | **Intervention Design Objectives** | **Key Features** |
| --- | --- | --- |
| Severe fatigue is common among youth with chronic health conditions. Often, treating the condition does not alleviate them from this fatigue. Youth suffer from fatigue-related impairments. | To increase the self-efficacy of youth in handling their fatigue to prevent impairments | Individualised trajectory in which youth have autonomy   - Personalisation of EMA-surveys - Personal insight into fatigue and associated factors (biological, psychological, social) - Shared decision-making on lifestyle goals - Self-experimenting with lifestyle goals |
| If paediatricians have ruled out a treatable medical cause, there are ample limited low-threshold tools to further support their patients. | To provide an accessible treatment (in cost & acceptability) option for preventing severe impairments due to fatigue | Cost:   - Secondary prevention of severe impairments would lead to long-term health care cost reduction. - Large part of intervention involves mHealth, reducing man-hours.   Acceptability:   - Transdiagnostic hospital-wide care provision - Guided by a specialised nurse, focusing on broad spectrum of modifiable factors (not only on psychological) |

Abbreviations: ESM is experience sampling methodology, mHealth is mobile health.

### Supplementary Table 2b. Booster’s Logic Model

Booster intervention is a behavioural change intervention. Booster’s logical model is based on the COM-B system, a framework describing behaviour as the result of three interacting conditions: capability, opportunity, and motivation.^5^ The **opportunity** to change is created by personal insight into fatigue and associated factors. The **capability** to change is enlarged via the support of the Booster app with the Experiment module, with goal setting based on the implementation intention^6^ and self-regulation strategies.^7^ Patients are primarily motivated due to the disabilities they already encounter. Booster enlarges and sustains this motivation targeting the basic psychological needs for autonomy, relatedness and competence, as described by the Self-Determination Theory.^8,9^ Booster’s logic model evolved during the participatory design process (Suppl. Table 2c). Highlighted in blue in Suppl. Table 2b: intervention processes new or changed from PROfeel to Booster. The PROfeel app’s functionality was the intensive ESM-surveys. The app had a role in the Measurement Period only. Its style was simple and plain, without colour. The major changes in the mHealth component are the following; gamification to support motivation for intensive and daily ESM, the incorporation of the Experiment Module, and an adolescent-friendly style.

| **Aim**  Goal of the intervention | **Intervention targets**  Behavioural/emotional and social processes impacting outcomes | **Intervention process**  Translation of processes into intervention | **App design features**  Translation of processes into mobile application | **Mechanisms of change**  Underlying theoretical constructs for intervention | **Key outcomes**  Changes aimed for |
| --- | --- | --- | --- | --- | --- |
| Early intervention of fatigue to avert fatigue progression and prevent fatigue-related impairments in adolescents (12 to 18 years) with a chronic health condition | **Opportunity** to change fatigue experience and perception | - Personal BPS model in Start C.^a^ - ESM - Insight C. | - Personalised intensive ESM-surveys^b^ in Measurement P. - Booster report | - Insight into personal fatigue-related factors - Concrete actionable lifestyle goals | Short term   - Increase in FSE   Long term   - Decrease fatigue - Increase participation - Increase QoL |
|  | **Capability** to change lifestyle factors | - Goal setting in Insight C. - Focus on experimenting with goals ^c^ - Self-regulation strategies | Experiment module ^c^:   - Implementation intention - Progress tracking of goal attainment and outcomes via daily ESM progress monitor - Feedback on goal attainment | Increased goal attainment skills |  |
|  | **Motivation** to change | Fulfil basic psychological needs for: |  | Sustained motivation for ESM and lifestyle experiments according to Self-Determination Theory |  |
|  |  | - Autonomy | 🡪 Personalisation^b^, game, diary, dream |  |  |
|  |  | - Relatedness (e.g., blended care^a^) | 🡪 Avatar, positive messages, diary |  |  |
|  |  | - Competence | 🡪 Progress tracking, awards |  |  |

^a^The PROfeel Start Conversation was conducted by a paediatrician specialised in fatigue. To work towards a less labour-intensive Booster, this step was omitted and replaced by an explanation of the BPS model by the investigator.

^b^ESM-items were revised to contain more options to personalise the items and give more specific answers to items (e.g., for current location).

^c^In the Insight Conversation, the participant and the investigator set lifestyle goals. This was followed in PROfeel by a lifestyle change period. For Booster, the focus shifted from ‘change’ to ‘experiment’ to better reflect Booster as a self-experimentation trajectory,^10^ in which goals are not fixed but can be flexibly adapted by the participants without the involvement of the investigator. This self-experimentation consists of the following steps: 1) In the Measurement Period, participants *track* symptoms, thoughts, feelings and activities. 2) In the Insight Conversation, participants generate *hypotheses* on how to gain FSE based on the Measurement Period data, 3) which will be *tested* in the Experiment Period. While experimenting with lifestyle goals, participants keep ownership over the process by selecting, evaluating and adapting their goals. This way, goal attainment is personalised by participants to support their own needs and fit their contexts.^11^ Personalisation has been shown to increase adherence and effectiveness of mHealth interventions.^12^

Abbreviations: BPS is biopsychosocial, C. is Conversation, COM-B is capability, opportunity, motivation – behaviour, ESM is experience sampling methodology, FSE is fatigue-related self-efficacy, mHealth is mobile health, P. is Period, QoL is quality of life.

### Supplementary Table 2c. Participatory Design Process

From September 2022 to April 2024, the Booster intervention was developed. Until March 2023, the design process focused on empathising with users, defining the development direction and generating ideas. From June 2023 onwards, the focus shifted to alternating between ideation, and prototype development and testing. The most important development steps are listed in chronological order. Not shown in Suppl. Table 2c: all prototypes were also tested by research team members, externally consulted paediatric researchers, and game and design experts. Output of empathise, ideation and test steps was discussed within the development team. This consisted of a paediatrician, game researcher, psychologist, game designers and app developers to define development direction further. Highlighted in blue in Suppl. Table 2c: design steps in which children with a chronic health condition participated.

| **Time** | **Aim and Activity** | **Participants**  % female, age in years (median [IQR])) | **Method** | **Lessons Learned** |
| --- | --- | --- | --- | --- |
|  |  |  |  |  |
| September ’22 | Empathise with (parent of) adolescents with JIA that receive lifestyle advice for fatigue | N=40, 10% adolescents with JIA, 90% parents of patients with JIA. Convenience sample. | Focus groups (N=3, empathy mapping) | - Desire to be normal, participate - Another ‘to do’ for their health - Importance for child to see relevance of ESM surveys - Importance to see progress |
| October ‘22 | Explore methods for support of behavioural change | N=8, psychologists. | Interviews | - Preconditions: feasibility, motivation and concrete goal - Development ideas:   - Rewards, positive reinforcement   - Monitoring progress   - Personalisation   - Reminders |
| November ‘22 | Explore gamification options to increase motivation for intervention | N=6, members of eHealth Junior consortium (e.g., medical doctor, game designer, psychologist). | Brainstorm | - Ideas for theme and game mechanics - For game elements stay close to user’s intrinsic motivation |
| **Time** | **Aim and Activity** | **Participants**  % female, age in years (median [IQR])) | **Method** | **Lessons Learned** |
|  |  |  |  |  |
| October ’22 to February ‘23 | Empathise with users of PROfeel | N= 11, patients (91% F, 17.5yr [15.5 – 20.5]). | Interviews | - Value: insight in fatigue - Lifestyle change difficult - Independent use by patients |
| October ’22 to February ‘23 | Explore the value of PROfeel in clinical care, implementation strategies | N= 20, clinicians (e.g., paediatricians, nurses, psychologists, 70% F, 46yr [40-57]). | Interviews | Central Booster practitioner, e.g., specialised nurse |
| January ‘23 | Ideate on app elements for motivating for ESM and lifestyle change | N=10, adolescents with a chronic condition and (history of) fatigue (60% F, 14yr [13-16]). | Focus groups (N=3) | - Personalisation and feedback on progress are important - Game preferences age seemed dependent, younger adolescents: mini-game, older adolescents: diary |
| March ‘23 | Ideate on app elements for motivating for ESM and lifestyle change | N= 7, researchers human-centred design. | Focus group | - Shift focus in the goal attainment period from “change” to “experiment” - Enlarge autonomy of participant during intervention |
| June ‘23 | Test prototype 1 (digital prototype, including ideas for rewards, game, goal attainment module) | N=6, hospital child advisory board. | Focus group | - Make extra game mechanics optional, not obligatory - Sharing with peers or family not desired |
| **Time** | **Aim and Activity** | **Participants**  % female, age in years (median [IQR])) | **Method** | **Lessons Learned** |
|  |  |  |  |  |
| September ‘23 | Test prototype 2 (digital prototype, including game mechanics, diary, style options) | N=6, adolescents with a chronic condition and (history of) fatigue (50% F, 17yr [14-17]). | Think aloud interviews | - Style should not be hospital-like or disease-focused, but also calm. - Stickers for diary - Minimalistic endless runner game appreciated |
|  |  | N= 46, convenience sample, reached via media channels paediatric hospital and patient associations (70% F, range 12 to 18 yr). | Survey online | - Styles too busy - Preference for abstract style |
| October ‘23 | Test prototype 3 (app with measurement Module, including style, ESM and diary) | N=5, adolescents with a chronic condition and (history of) fatigue (60% F, 17yr [13-17]). | Interview after a 4-day test period of the app | - Style appreciated - Create more emoticons for the diary |
| January ‘24 | Test name for intervention, texts, style | N=8, hospital child advisory board. | Focus group & survey online | - Renamed Booster - Changes in avatar texts |
| April ‘24 | Test prototype 4 (app with Measurement and Experiment module, including game) | N=8, adolescents with a chronic condition and (history of) fatigue (75% F, 16.5yr [13-17]). | Interview after 7 to 14 days test period of the app | - Nice game, played it a bit, not addictive - Diary used by some, others ‘not my cup of tea’ - The progress monitor viewer was valued, but often not seen in the menu. - Participants did barely look at the ‘Dream’ option |

Abbreviations: IQR is interquartile range, F is female, yr is years.

### Supplementary Table 2d. Booster App Functionalities

Detailed description of Booster app functionalities and their rationales. If specific comments were made by patients during the participatory design process (Suppl. Table 2c), these are provided in the column “Patient Input”. Functionalities are grouped in functionalities always present (highlighted in purple), functionalities only present in the Measurement Module (highlighted in blue), and functionalities only present in the Experiment Module (highlighted in green).

| **General – Functionalities Present in Each Booster Intervention Stage (Suppl. Fig. 2 to 3)** | | | |
| --- | --- | --- | --- |
| Functionality | Description | Goal/Rationale | Patient Input |
| Style (Suppl. Fig. 2 to 5) | Colourful but calm. Sky/space themed. |  | - Create a non-‘hospital-like’ environment - Give a happy feeling, does not refer directly to fatigue - Prevent tiredness due to too busy style - Abstract space theme liked most across ages |
| Game (Suppl. Fig.2) | The user pilots the avatar over an increasingly fast side-scrolling track by tapping the screen to avoid obstacles. In the game store, the participant can use the points to buy new track elements, to create, for example, a longer, easier or prettier track. | Autonomy :   - Personalisation of track - Ability to use the app on own initiative   Competence:   - Improve game skills   Track that grows symbolises the duration of the Booster journey of the user; obstacles symbolise the obstacles in real life. | - Nice distraction but not addictive - Rewarding |
| Avatar (Suppl. Fig.2A-B) | At home screen and in game, also depicted in some awards. Happy yellow human-like figure with a ‘sun’ head. Greets the user, and uses the nickname of the user. | Relatedness: personal connection  Symbolises energy |  |
| Progress monitor | Daily ESM survey that asks the user to reflect upon the day | Competence: Monitoring | Insightful to end each day with |
| Functionality | Description | Goal/Rationale | Patient Input |
| Points & awards (Suppl. Fig. 3A-B) | For each questionnaire answered, the user receives points. For specific achievements, like a streak, or a period of high compliance, the user receives bonus points and an award, accompanied by positively phrased text. | Competence: give the feeling of mastery | Motivating to receive points and badges |
| Daily diary (Suppl. Fig. 3C) | The user can add one dairy entry per day, by writing text and/or choosing a sticker. Entries can be adapted later.  The diary page gives an overview of all entrees. | Relatedness: personal connection  Autonomy:   - Optional - Free space, choice for adding text and/or colourful stickers - Not shared with research team | - Sticker option, since writing can be difficult if you are tired. - Monitor how you are feeling.   Having to fill out that you are feeling bad multiple times a day can be confronting, for this reason wish to have an option to fill out what you are happy with/grateful for. |
| Personalisation of surveys (Suppl. Fig 3D) | Personalisation of ESM items by adapting phrasing of items, and adding relevant items  Personalisation of ESM schedules for intensive ESM (measurement period) and daily ESM in progress monitor. | Autonomy: provide choice |  |
| Contact investigator (Suppl. Fig 3E) | Once a week a question is posed in the daily progress monitor whether the user wants to contact the investigator with questions. Moreover, contact details to reach out to the investigator are known to the user. | Relatedness: provide opportunities for support |  |
| Positive messages | All messages received, irrespective of compliance or goal attainment, are positively and supportively formulated. | Relatedness: unconditional regard |  |

| **Measurement Period Specific – Functionalities Present in Measurement Period Only (Suppl. Fig. 4)** | | | |
| --- | --- | --- | --- |
| Functionality | Description | Goal/Rationale | Patient Input |
| Daily survey overview (Suppl. Fig. 4A) | Home screen shows the surveys planned and completed for the day | Competence: monitoring of progress |  |
| Award overview (Suppl. Fig. 4B) | The obtained awards (with award picture), and progress on future awards (yellow bar filling) is shown. | Competence: monitoring of progress |  |
| Intensive ESM-surveys (Suppl. Fig. 4C-D) | 5-times daily short surveys that asks to reflect on the past 3 hours, called “Check-up”. | Needed for Booster report (overview of fatigue fluctuations and associated lifestyle factors) | Filling out gives insight |
| **Experiment Period Specific – Functionalities Present in Experiment Period Only (Suppl. Fig. 5)** | | | |
| Functionality | Description | Goal/Rationale | Patient Input |
| Report | The lifestyle report can be viewed in the app. | Autonomy: provide meaningful rationale for change, elicit perspectives on condition and behaviour. |  |
| Progress overview (Suppl. Fig. 5A) | Outcomes of the daily ESM progress monitor are shown. | Competence: monitor progress | Noticing positive effect helps |
| Active Goal on main screen with goal attainment overview (Suppl. Fig. 5B) | Reminder for active goal and show previously achieved goals. | Competence: monitor progress  Show perseverance |  |
| Goal overview (Suppl. Fig. 5C-D) | The user fills in their active goal with an implementation intention. Multiple goals can be added, however, only one goal can be active. | Competence: clear and concrete plan of action |  |
| Dream (Suppl. Fig. 5E) | a larger overarching goal to strive for (e.g., fully participating at school or having fun with friends), that may motivate to work on the smaller lifestyle goals. | Autonomy: explore life aspirations and values, provide meaningful rationale |  |
| Functionality | Description | Goal/Rationale | Patient Input |
| Motivating messages | The participant receives motivating messages and bonus points at time-related milestones.  In messages, references are amongst other things made to the active goal, to the personal dream, and the progress overview. | Competence: promote self-monitoring  Relatedness: unconditional regard |  |
| Goal attainment feedback | Based on goal evaluation end of the day, the participant receives advice on how the ‘tweak’ their goals, by making them easier, or more challenging to reach. | Autonomy: give suggestions, non-controlling language, encourage users to experiment.  Competence: assist in setting the optimal challenge |  |


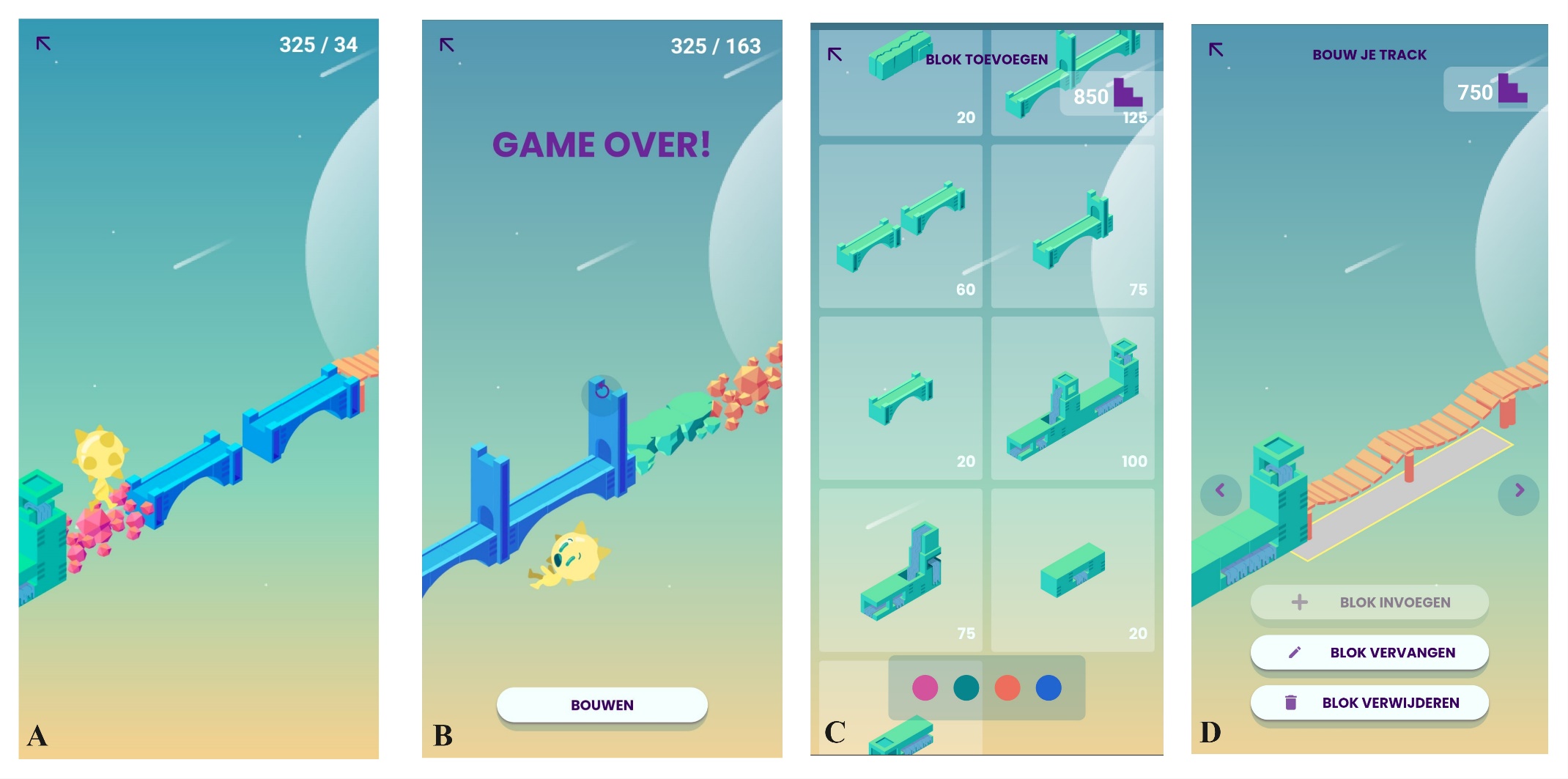


Supplementary Figure 2. The game. The user pilots the track with the avatar (A & B), can buy new building blocks in the game store (C) to personalise the track (D).


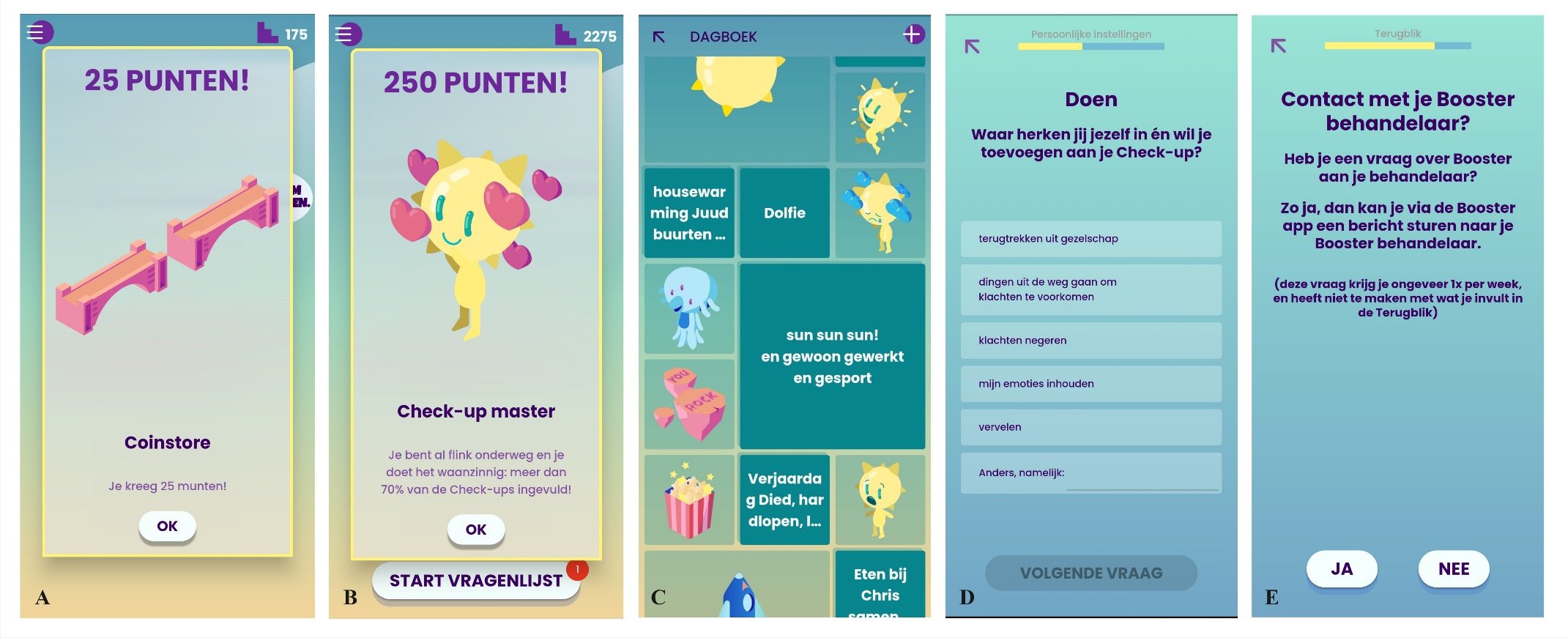


Supplementary Figure 3. Other general functionalities. Points (A), awards (B), daily diary (C), intensive ESM survey personalisation (D) and contact investigator (E).


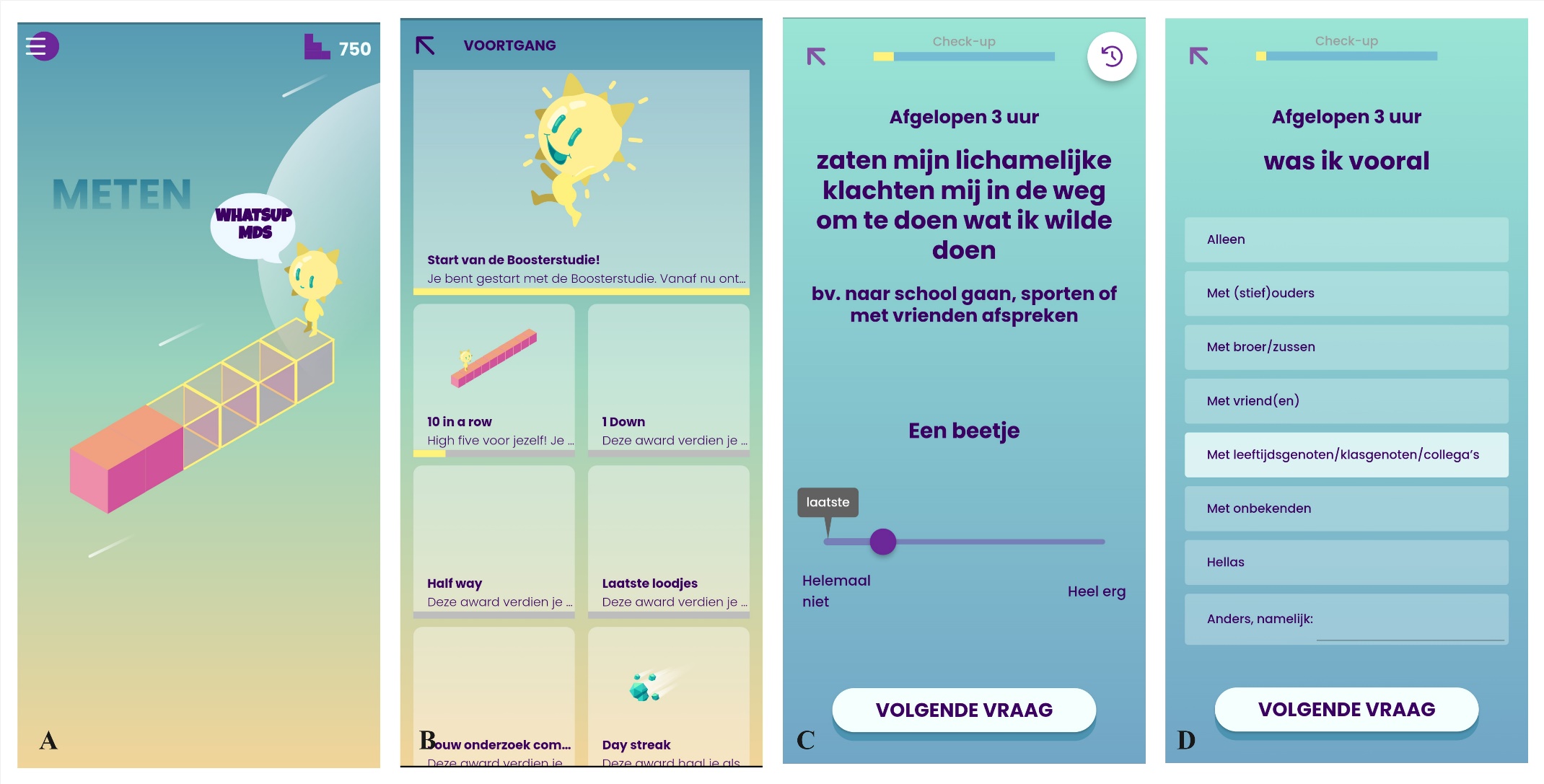


Supplementary Figure 4. Measurement Period-specific functionalities. Home screen with daily survey overview (A), award overview (B), and intensive ESM-surveys (C & D)
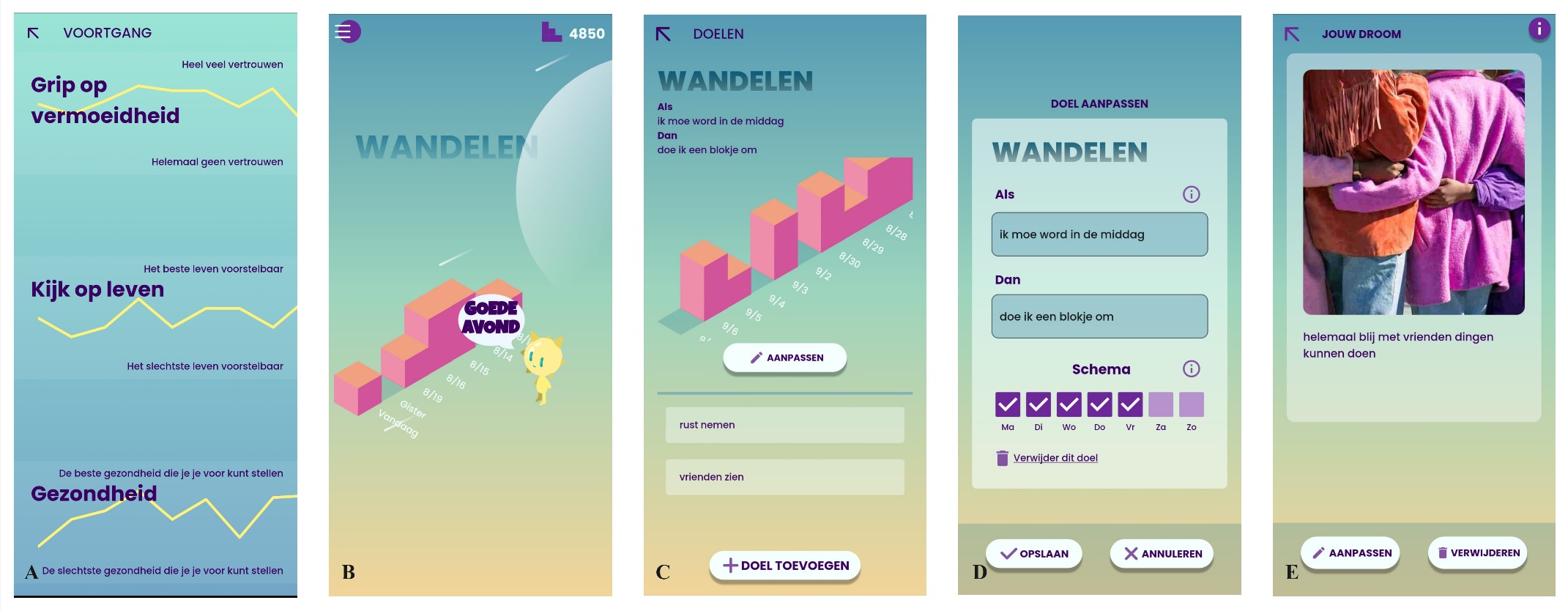
Supplementary Figure 5: Experiment Period specific functionalities. **P**rogress overview (A), home screen with active goal and goal attainment (B), goal overview and adaptation ( C&D), and Dream (E).

## Supplementary Table 3. Participant Timeline

Study timeline and activities are described. Highlighted in blue: duration of the daily ESM progress monitor.

| **Week** | **Activity** | **Description** |
| --- | --- | --- |
| 0 | T0  Baseline assessment | Participant fills out baseline questionnaires from home, and installs the Booster app on their phone. The daily ESM progress monitor starts and will continue until T3. |
| 0-2 | Preparatory stage | Participant completes the daily ESM progress monitor. The preparatory stage duration is randomised between ten and eighteen days. |
| 2 | T1  Start  Conversation | Investigator explains the biopsychosocial model to the participant. Guided by investigator, the participant personalises the intensive ESM-survey in the Booster app. |
| 2-6 | Measurement Period | Participant receives five personalised ESM-surveys a day via the Booster app. Each survey asks to reflect on the last three hours and contains items on symptoms, thoughts, feelings and activities. |
| 6 | T2  Insight  Conversation | Participant receives the Booster report with personal fatigue insight from the investigator. Via shared-decision making, personal lifestyle goals are set. |
| 6-18 | Experiment  Period | Participant experiments with one personal lifestyle goal at a time, supported by the Booster app. |
| 18 | T3  Evaluation  Conversation | Participant reflects on the effects of Booster, guided by the investigator. Afterwards, a semi-structured qualitative interview is conducted to assess the effect and user experience of Booster in general and the app in specific. The participant fills out follow-up questionnaires. |
| 31 | T4 | Participant fills out follow-up questionnaires. |
| 57 | T5 | Participant fills out follow-up questionnaires. T5 is the end of study participation. |

Abbreviation: ESM is experience sampling method.

## Supplementary Table 4. Example of a Biopsychosocial Model of Fatigue

|  | **Biological** | **Psychological** | **Social** |
| --- | --- | --- | --- |
| **Predisposing** | JIA, medication | Depression | Coping strategy of family with JIA |
| **Precipitating (inciting)** | Sport injury, viral infection | Stress | School pressure |
| **Perpetuating (sustaining)** | Irregular sleep and eating routine | The feeling of no control | Social isolation |

Abbreviation: JIA is juvenile idiopathic arthritis.

## Supplementary Table 5. Items in ESM-survey of Measurement Period

Participants receive their personalised intensive ESM-survey five times daily during the Measurement Period. The four sleep items are only posed in the first survey of the day. The participant personalises the survey in the Start Conversation, by doing the following: 1) add up to two extra items on specific physical symptoms 2) choose the exact phrasing of items from a list or create their own, and 3) add one extra item (not shown in table). Total survey length ranges from 21 (first of the day, 3 extra items) to 14. Personalisation options are displayed between [brackets], and personalised questions are highlighted in blue.

| **Construct (number of items)** | **Item** | **Answer scale** |
| --- | --- | --- |
| Sleep  (4) | - Last night, I went to bed at: | Hours:minutes |
|  | - Today, I got out of bed at: | Hours:minutes |
|  | - Last night, I slept from the time I went to bed: | VAS: 0 (0 hours) - 16 (16 hours) |
|  | - I felt rested upon waking up | VAS: 0 (not at all) to 100 (very much) |
| Physical symptoms  (3-5) | - In the past 3 hours, my physical symptoms were | VAS: 0 (not severe at all) - 100 (very severe) |
|  | - In the past 3 hours, my physical symptoms interfered with doing the things I wanted to do (e.g., going to school, exercising, seeing friends) | VAS: 0 (not at all) to 100 (very much) |
|  | - In the past 3 hours, I felt tired | VAS: 0 (not at all) to 100 (very much) |
|  | - In the past 3 hours I had [a headache]   Examples of other options: back pain/ nausea/dizziness/concentration problems/memory problems | VAS: 0 (not at all) to 100 (very much) |
| Behaviour  (4) | - In the past 3 hours, I did the following [withdrawing from company]   Examples of other options: avoiding things to prevent symptoms/ignoring symptoms/suppressing my emotions/feeling bored | VAS: 0 (not at all) to 100 (very much) |
|  | - The last 3 hours I was physically active (e.g., cycled, strolled, exercised) |  |
|  | - In the past 3 hours, I have been lying down/resting/sleeping during the day |  |
|  | - In the past 3 hours, I had to use my brain, for example, thinking hard or studying for school |  |
| Thoughts  (1) | - In the past 3 hours, I thought/felt the following: [stress from wanting or having to do too much]   Examples of other options: worrying about my symptoms/worrying in general/being preoccupied with what others think of me. | VAS: 0 (not at all) to 100 (very much) |
| Feelings  (2) | - In the past 3 hours, I felt [happy]   Examples of other options: cheerful/energetic/proud | VAS: 0 (not at all) to 100 (very much) |
|  | - In the past 3 hours, I felt [angry]   Examples of other options: miserable/anxious/frightened/sad |  |
| Social context (4) | - In the past 3 hours, I was mostly | Multiple choice   - At home (in my bedroom) - At home (not in my bedroom) - At someone else's home - At school/work - Outside - On the road - Other, [open field] |
|  | - In the past 3 hours, I was mostly | Multiple choice   - Alone - With parents - With siblings - With friend(s) - With peers/classmates/colleagues - With strangers - Other, [open field] |
|  | - In the past 3 hours, I had pleasant contact with people | Yes/no |
|  | - In the past 3 hours, I had unpleasant contact with people | Yes/no |

Abbreviation: VAS is visual analogue scale.

## Supplementary Table 6. Proposed Covariates Measured at Baseline and Follow-up

| **Focus** | **Construct** | **Questionnaire (reference)** | **Items (N)** | **Timing** |
| --- | --- | --- | --- | --- |
| Potential covariates reflecting constructs measured in progression monitor (i.e., outcomes) | Fatigue-related self-efficacy | Self-Efficacy Scale-28 (SES-28)^13^ | 7 | Baseline and follow-up |
|  | Fatigue | - Pediatric short fatigue questionnaire (pSFQ)^14^ - PEDsQL-Multidimensional Fatigue Scale (MFS)^15^ | 4  24 |  |
|  | Quality of life | - PEDsQL- Generic Core Scales (GCS)^16^ - Child Health Utility Index 9D (Chu9d)^17,18^ - EuroQol Five Dimensions Health Questionnaire Youth (EQ-5D-Y-3L)^19^ - NRS Cantril ladder^20^ | 23  9  6  1 |  |
|  | Participation | School or work absence ^2^ | 1 |  |
| Potential covariates | Internalising symptoms | Revised Child Anxiety and Depression Scale (RCADS)^21,22^ | 25 | Baseline |
|  | Pain | NRS pain^23^ | 1 |  |
| Potential goal-related covariates | Illness identity | Illness Identity Questionnaire (ILQ) – fatigue^24^ | 25 | Baseline and follow-up |
|  | Mastery | Pearlin Schooler Mastery Scale^25^ | 7 |  |
|  | Physical activity | [Physical Activity Questionnaire for Adolescents (PAQ-A)^26^](https://fcon_1000.projects.nitrc.org/indi/cmi_healthy_brain_network_old/assessments/paq-a.html) | 9 |  |

Abbreviations: PEDsQL is Pediatric Quality of Life Inventory, NRS is numeric rating scale.

## Supplementary File 1. Construction and Validation of a Single Item for Measuring Fatigue-related Self-efficacy

Self-efficacy is defined as the belief in one’s capabilities to influence events affecting their life.^27^ Fatigue-related Self-efficacy (FSE) is one's confidence in dealing with fatigue.^13^ A single item to measure FSE was constructed to minimise the daily ESM progression monitor length. This qualitative study measures the validity of the single FSE item.

***Methods***

Respondents were asked to answer the item out loud, answer the item, explain the rationale for their response and paraphrase the item. One investigator conducted the interviews and used probing questions inspired by the Response Process Evaluation Method.^28^ Interviews were audio recorded and transcribed verbatim. Two investigators (psychologist and medical doctor) independently assessed respondent’s understanding. In case < 80% of the respondents understood the item, it was revised in group discussions between the two investigators and other research team members (paediatricians, psychologist).

***Results***

Three interview rounds with a total of seventeen respondents were conducted until face validity stabilised with 60% of respondents understanding the item (Suppl. File 1 Table 1). Respondents were adolescents aged 13 to 17 years with a chronic health condition. All had (a history of) severe fatigue.

For the first iteration, the FSE item as administered in an earlier PROfeel trial was evaluated.^2^ This FSE item was inspired by the first item of the Self-Efficacy Scale-28 (SES-28), a seven-item questionnaire on FSE.^13^ Interpretation of the two independent researchers on understanding did not match apart from one respondent. One researcher had compared responses to the literal meaning of the question (e.g., grip over fatigue as taking a nap), which most children had understood. The other researcher compared responses to the meaning of FSE as was targeted by the Booster intervention (e.g., grip over fatigue as not letting it interfere with activities), which most children did not understand. The interpretation discrepancy of the question between even two researchers, familiar with FSE showed that the question should be adapted.

For the second iteration, the item was inspired by the first question of the self-efficacy for Managing Chronic Diseases 6-item scale.^29^ Five out of eight respondents understood the item. Others overlooked the ‘trust’ part of the item.

For the third iteration, the item was shortened, and ‘trust’ was emphasised by incorporating it into the answering scale. Two versions of the item were created: one using the verb ‘trust’ and the other ‘believe’. Only one respondent understood the item immediately. Two respondents realised they had not read the item carefully and corrected themselves after explaining their chosen FSE level. Two others required more guidance from the investigator to grasp the meaning. All respondents preferred the verb ‘believe’, citing familiarity as the main reason. No suggestions were made to clarify or simplify the item further. Although the targeted 80% comprehension was not achieved, the item from the third iteration using ‘believe’ was selected. In a research team discussion, no alternative phrasing was found to better balance clarity and simplicity.

Across the three iterations, respondents noted that FSE was not a concept they typically considered, though they found it meaningful. Some spontaneously remarked that it was a helpful question to answer.

**Conclusion**
For many adolescents, the FSE item and corresponding construct were not immediately understood. Understanding improved for most following reflective prompts, allowing them to give concrete examples from their daily lives illustrating low or high FSE. Therefore, it was decided to introduce and explain the FSE item at the start of the study (T0) using the same methodology as in the item validation process. This approach ensures proper understanding from the outset, preventing response shifts over time.

**Supplementary File 1 Table 1.** Validation of single-item for fatigue-related self-efficacy.

| **Iteration** | **Item**  (Dutch, *English translation*, [ends of answering scale]) | **Understood**  (n/n total (%)) |
| --- | --- | --- |
| 1 | Vandaag dacht ik dat ik zelf mijn vermoeidheid kon beïnvloeden [Helemaal niet, Heel erg]  *Today I thought I could influence my fatigue [Not at all, Very much]* | 0/4 (0%) |
| 2 | Vandaag vertrouwde ik dat  **ik kon voorkomen dat vermoeidheid mij dwars zat**  bij de dingen die ik deed [Helemaal niet, Heel erg]  *Today I trusted that*  ***I could prevent fatigue from bothering me***  *in the things I did [Not at all, Very much]* | 5/8 (63%) |
| 3 | Vandaag  Geloofde/vertrouwde ik  dat ik kon voorkomen dat vermoeidheid mij dwars zat [Geloofde/vertrouwde ik helemaal niet, Geloofde/vertrouwde ik helemaal]  *Today*  *I believed/trusted*  *that I could prevent fatigue from bothering me [I didn’t believe/trust it at all, I fully believed/trusted it]* | 3/5 (60%) |

## Supplementary File 2. Usability Questionnaire

The usability questionnaire will be completed via the Booster app. Each participant will fill it out twice: 1) after the Measurement Period, just before the Insight Conversation, and 2) at the end of the Experiment Period, just before the Evaluation Conversation. Questions specific to the first or second timepoint are indicated with [M] and [E], respectively. The estimated time to complete the questionnaire is ten minutes.

**Usability**

1. Did you received enough information or explanation to use the Booster app? [Yes/No]
   - If No is selected: What information did you miss? […]
2. What rating would you give the Booster app in the following areas?
   - Helpful [1, Not at all helpful – 10, Very helpful]
   - Fun [1, Not at all fun – 10, Very fun]
   - Clear [1, Not at all clear – 10, Very clear]
   - Appearance/style [1, Not nice at all – 10, Very nice)
3. What overall rating would you give the Booster app? [1-10]
4. Would you like to use the Booster app again in the future? [Yes/No]
   - Yes: Why?
   - No: Why not?
5. How likely are you to recommend the Booster app to a friend who often feels tired? [1, Not at all likely – 10, Very likel]
   - Why did you give this score? […]

**Motivation**

1. I choose how I use the Booster app [1, Not at all – 10, Completely]
2. The Booster app motivates me to do things that are very difficult [1, Not at all – 10, Completely]
3. Version [E] or [M]

**[E]** How motivating were the following Booster components for you to keep up with your Check-ups? [1, Not at all motivating – 10, Very motivating]

- Playing the game
- Building my track in the game
- The points I received for the Check-ups
- The positive messages in the app
- The diary
- My progress (award images)
- Conversations with the Booster coach

**[M]** How motivating were the Booster components for you to keep experimenting with your goals? [1, Not at all motivating – 10, Very motivating]

- Playing the game
- Building my track in the game
- The points I received for the daily progress monitor
- The positive messages in the app
- The diary
- Conversations with the Booster coach
- My progress (insight into what you have filled in)
- My report
- My dream

**Effect**

1. The conversations with the Booster coach were valuable.
2. What is the effect of Booster on you? [multiple options can be selected]
   - I gained insight into my complaints.
   - I can better accept my complaints.
   - I have more control over my complaints.
   - I have fewer complaints.
   - I feel better.
   - I have become more active.
   - Booster did not help me with anything.
   - Other, namely…
3. [M] The Booster app helped me believe that I can prevent fatigue from hindering me [1, Not at all helpful – 10, Very helpful]

[E] Booster (app & conversations) helped me believe that I can prevent fatigue from hindering me [1, Not at all helpful – 10, Very helpful]

1. What helped you believe that? (options with [E] only shown at second time point)

- Personalisation of the Booster app in the Start Conversation
- Filling in Check-ups
- Filling in the daily progress monitor
- Diary
- [E] Report in the app
- [E] Progress (yellow graphs) in the app
- [E] Achieving my goals
- [E] conversations with Booster coach

1. What do you like best about Booster? […]
2. What do you like least about Booster? […]
3. What would you like to change or add to the Booster app? […]

## Supplementary File 3. Semi-structured Interview – topic guide

Overview of interview topics. Text in italics represents subtopics that can be addressed in follow-up questions to deepen understanding of the first response.

**Part 1: Understanding the effect of the intervention**

1. Effect of Booster
   1. What has changed? What is different now compared to before you started?
   2. What has remained the same?

*Control over fatigue / Fatigue / Health / Satisfaction*

1. Cause of change

*Health, environment, events, app, motivation*

1. Cause of lack of change

*Health, environment, events, app, motivation*

**Part 2: Evaluate the Booster intervention in general and the Booster app specifically**

1. Experience with Booster
2. Motivation for Booster intervention
3. Effect of Booster (elements)

*Start Conversation / Measurement Period / Insight conversation / Experiment Period*

1. Feasibility and usability
   1. Measurement Period
   2. Experimentation Period

*Helping factors and undermining factors*

*e.g., (social) environment, app use, type of goal*

1. Use of Booster app
   1. Measurement Period
   2. Experiment Period

*(De)motivating elements*

1. Feedback on Booster intervention
   1. Improvement opportunities
   2. Strengths

# Supplementary References

1. Chan AW, Tetzlaff JM, Gøtzsche PC, et al. SPIRIT 2013 explanation and elaboration: guidance for protocols of clinical trials. BMJ 2024; doi: 10.1136/bmj.e7586.

2. Vroegindeweij A, Swart JF, Houtveen J, et al. Identifying disrupted biological factors and patient-tailored interventions for chronic fatigue in adolescents and young adults with Q-Fever Fatigue Syndrome, Chronic Fatigue Syndrome and Juvenile Idiopathic Arthritis (QFS-study): study protocol for a randomized controlled trial with single-subject experimental case series design. Trials 2022;23(1):683; doi: https://doi.org/10.1186/s13063-022-06620-2.

3. Vroegindeweij A, Houtveen J, Lucassen DA, et al. Individual outcomes after tailored versus generic self-management strategies for persistent fatigue in youth with a fatigue syndrome or rheumatic condition: A multiple single-case study. Br J Health Psychol 2024; doi: 10.1111/bjhp.12722.

4. Vroegindeweij A, Wulffraat NM, Van De Putte EM, et al. Targeting persistent fatigue with tailored versus generic self-management strategies in adolescents and young adults with a fatigue syndrome or rheumatic condition: A randomized crossover trial. Br J Health Psychol 2023; doi: 10.1111/bjhp.12711.

5. Michie S, van Stralen MM, West R. The behaviour change wheel: A new method for characterising and designing behaviour change interventions. Implementation Science 2011;6(1); doi: 10.1186/1748-5908-6-42.

6. Gollwitzer PM, Sheeran P. Implementation Intentions and Goal Achievement: A Meta-Analysis of Effects and Processes. Adv Exp Soc Psychol 2006;38:69–119; doi: 10.1016/S0065-2601(06)38002-1.

7. Gillebaart M, Brummelman J, de Ridder D, et al. Study Protocol of the Ten Years Up Project: Mapping the Development of Self-Regulation Strategies in Young Adults Over Time. Front Psychol 2021;12; doi: 10.3389/FPSYG.2021.729609.

8. Teixeira PJ, Marques MM, Silva MN, et al. A classification of motivation and behavior change techniques used in self-determination theory-based interventions in health contexts. Motiv Sci 2020;6(4):438–455; doi: 10.1037/mot0000172.

9. Ryan R, Deci EL. Self-Determination Theory Basic Psychological Needs in Motivation, Development, and Wellness. Guilford Press: New York; 2017.

10. Karkar R, Zia J, Vilardaga R, et al. A framework for self-experimentation in personalized health. Journal of the American Medical Informatics Association 2016;23(3):440–448; doi: 10.1093/jamia/ocv150.

11. Lee J, Walker E, Burleson W, et al. Self-Experimentation for Behavior Change: Design and Formative Evaluation of Two Approaches. In: Conference on Human Factors in Computing Systems - Proceedings Association for Computing Machinery; 2017; pp. 6837–6849; doi: 10.1145/3025453.3026038.

12. Jakob R, Harperink S, Rudolf AM, et al. Factors Influencing Adherence to mHealth Apps for Prevention or Management of Noncommunicable Diseases: Systematic Review. J Med Internet Res 2022;24(5); doi: 10.2196/35371.

13. Bleijenberg G, Bazelmans E, Prins J. Self-­efficacy Schaal (SES). In: Chronisch Vermoeidheidssyndroom. Praktijkreeks Gedragstherapie. Deel 13 Bohn Stafleu van Loghum; 2001; pp. 0–106.

14. Nap-van der Vlist MM, Vroegindeweij A, Hoefnagels JW, et al. Paediatric short fatigue questionnaire, a 4-item fatigue questionnaire for children. J Psychosom Res 2023;165:111130; doi: 10.1016/j.jpsychores.2022.111130.

15. Gordijn SM, Cremers EMP, Kaspers GJL, et al. Fatigue in children: reliability and validity of the Dutch PedsQL^TM^ Multidimensional Fatigue Scale. Qual Life Res 2011;20(7):1103–1108; doi: 10.1007/S11136-010-9836-9.

16. Engelen V, Haentjens MM, Detmar SB, et al. Health related quality of life of Dutch children: Psychometric properties of the PedsQL in the Netherlands. BMC Pediatr 2009;9:68; doi: 10.1186/1471-2431-9-68.

17. Stevens KJ. Working with children to develop dimensions for a preference-based, generic, pediatric, health-related quality-of-life measure. Qual Health Res 2010;20(3):340–351; doi: 10.1177/1049732309358328.

18. Rowen D, Mulhern B, Stevens K, et al. Estimating a Dutch Value Set for the Pediatric Preference-Based CHU9D Using a Discrete Choice Experiment with Duration. Value in Health 2018;21(10):1234–1242; doi: 10.1016/j.jval.2018.03.016.

19. Ravens-Sieberer U, Wille N, Badia X, et al. Feasibility, reliability, and validity of the EQ-5D-Y: Results from a multinational study. Quality of Life Research 2010;19(6):887–897; doi: 10.1007/s11136-010-9649-x.

20. Cantril H. The Pattern of Human Concern. Rutgers University Press: New Brunswick; 1965.

21. Kösters MP, Chinapaw MJM, Zwaanswijk M, et al. Structure, reliability, and validity of the revised child anxiety and depression scale (RCADS) in a multi-ethnic urban sample of Dutch children. BMC Psychiatry 2015;15(1); doi: 10.1186/s12888-015-0509-7.

22. Ebesutani C, Reise SP, Chorpita BF, et al. The Revised Child Anxiety and Depression Scale-Short Version: Scale reduction via exploratory bifactor modeling of the broad anxiety factor. Psychol Assess 2012;24(4):833–845; doi: 10.1037/a0027283.

23. Rosier EM, Iadarola MJ, Coghill RC. Reproducibility of pain measurement and pain perception. Pain 2002;98:205–216; doi: 10.1016/s0304-3959(02)00048-9.

24. Oris L, Rassart J, Prikken S, et al. Illness identity in adolescents and emerging adults with type 1 diabetes: Introducing the illness identity questionnaire. Diabetes Care 2016;39(5):757–763; doi: 10.2337/dc15-2559.

25. Pearlin LI, Schooler C. The Structure of Coping. J Health Soc Behav 1978;19(1):2–21.

26. Bervoets L, Van Noten C, Van Roosbroeck S, et al. Reliability and Validity of the Dutch Physical Activity Questionnaires for Children (PAQ-C) and Adolescents (PAQ-A). Archives of Public Health 2014;72(1); doi: 10.1186/2049-3258-72-47.

27. Bandura A. Self-Efficacy: The Exercise of Control. WH Freeman: New York; 1997.

28. Wolf MG, Ihm E, Maul A, et al. The Response Process Evaluation Method. Preprint 2023.

29. Ritter PL, Lorig K, Laurent DD. Characteristics of the Spanish- and English-Language Self-Efficacy to Manage Diabetes Scales. Diabetes Educator 2016;42(2):167–177; doi: 10.1177/0145721716628648.
